# Tracking the Stability of Clinically Relevant Blood Plasma Proteins with Delta-S-Cys-Albumin—A Dilute-and-Shoot LC/MS-Based Marker of Specimen Exposure to Thawed Conditions

**DOI:** 10.1016/j.mcpro.2022.100420

**Published:** 2022-09-28

**Authors:** Erandi P. Kapuruge, Nilojan Jehanathan, Stephen P. Rogers, Stacy Williams, Yunro Chung, Chad R. Borges

**Affiliations:** 1 School of Molecular Sciences, Arizona State University, Tempe, Arizona, USA; 2 The Biodesign Institute at Arizona State University, Tempe, Arizona, USA; 3 College of Health Solutions, Arizona State University, Phoenix, Arizona, USA

**Keywords:** plasma, serum, Biobanking, quality control, thawed, CVD, cardiovascular disease, EGF, Epidermal Growth Factor, GI, gastrointestinal, MAP, multi-analyte profile, P/S, plasma/serum, PAV, pre-analytical variable

## Abstract

Biomolecular integrity can be compromised when blood plasma/serum (P/S) specimens are improperly handled. Compromised analytes can subsequently produce erroneous results—without any indication of having done so. We recently introduced an LC/MS-based marker of P/S exposure to thawed conditions called ΔS-Cys-Albumin which, aided by an established rate law, quantitatively tracks exposure of P/S to temperatures greater than their freezing point of −30 °C. The purposes of this study were to (1) evaluate ΔS-Cys-Albumin baseline values in gastrointestinal cancer patients and cancer-free control donors, (2) empirically assess the kinetic profiles of ΔS-Cys-Albumin at 23 °C, 4 °C, and −20 °C, and (3) empirically link ΔS-Cys-Albumin to the stability of clinically relevant proteins. ΔS-Cys-Albumin was measured at ≥ 9 different time points per exposure temperature in serum and K_2_EDTA plasma samples from 24 separate donors in aliquots kept separately at 23 °C, 4 °C, and −20 °C. Twenty-one clinically relevant plasma proteins were measured at four time points per temperature *via* a multiplexed immunoassay on the Luminex platform. Protein stability was assessed by mixed effects models. Coordinated shifts in stability between ΔS-Cys-Albumin and the unstable proteins were documented by repeated measures and Pearson correlations. Plasma ΔS-Cys-Albumin dropped from approximately 20% to under 5% within 96 h at 23 °C, 28 days at 4 °C, and 65 days at −20 °C. On average, 22% of the 21 proteins significantly changed in apparent concentration at each exposure temperature (*p* < 0.0008 with >10% shift). A linear inverse relationship was found between the percentage of proteins destabilized and ΔS-Cys-Albumin (*r* = −0.61; *p* < 0.0001)—regardless of the specific time/temperature of exposure. ΔS-Cys-Albumin tracks cumulative thawed-state exposure. These results now enable ΔS-Cys-Albumin to approximate the percentage of clinically relevant proteins that have been compromised by incidental plasma exposure to thawed-state conditions.

## Highlights

• Baseline ΔS-Cys-Albumin in GI cancer patients and control donors determined.
• Twenty-four time courses for matched plasma/serum samples at 23 °C, 4 °C, and −20 °C.
• ΔS-Cys-Albumin linked to protein stability independent of temperature.
• Minimal differences existed between GI cancer patients and cancer-free donors.

## Graphical Abstract

**Figure:**
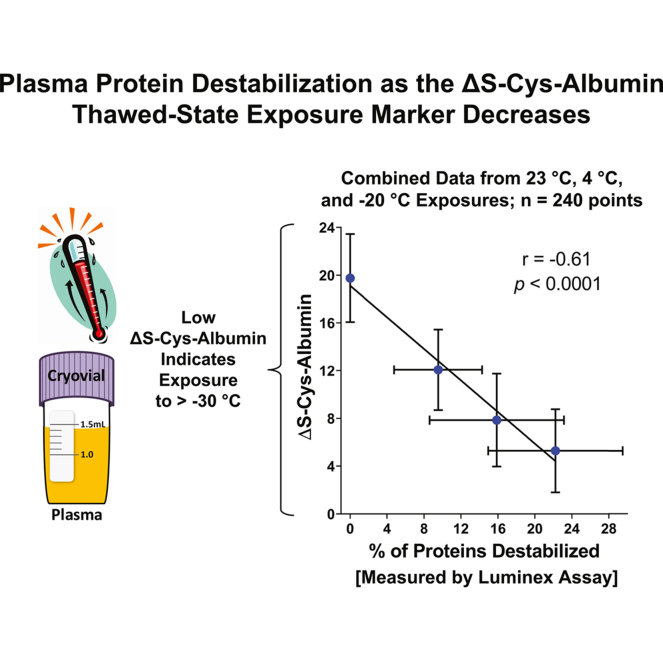

## In Brief

ΔS-Cys-Albumin is an endogenous marker of plasma and serum exposure to thawed conditions (> -30 °C). Baseline values of ΔS-Cys-Albumin in gastrointestinal cancer patients and cancer-free donors were determined. Time courses for 12 patients from each cohort were acquired at 23 °C, 4 °C, and −20 °C. ΔS-Cys-Albumin decay kinetics were then empirically linked to the stability of 21 proteins measured *via* Luminex assay. ΔS-Cys-Albumin was linearly correlated to the percentage of destabilized plasma proteins, regardless of exposure temperature.

As the acellular component of blood, plasma/serum (P/S) exchanges biological information in the form of biomolecules with every organ system in the body. Because it carries this wealth of information and can be noninvasively collected, P/S is one of the most common biospecimens employed in biomedical research. Moreover, P/S samples collected during clinical studies frequently carry potential research value well beyond that intended by the original research design and are therefore archived for future research purposes. Millions of P/S samples are currently stored in biobanks around the world awaiting withdrawal and subsequent queries on the molecular information they contain. The answers to these queries, however, reflect both *in vivo* and *ex vivo* biochemistry—meaning that if the latter has impacted the former, incorrect and potentially misleading information will be obtained. This can readily occur when pre-analytical variables (PAVs) are inadequately controlled (1, 2, 3, 4, 5, 6, 7, 8).

During sample collection, processing, transport, and storage, every biospecimen is exposed to PAVs. While the number of PAVs can be quite large (7, 8, 9, 10, 11, 12, 13), proper sample handling techniques can keep them tightly controlled, protecting *in vivo* biochemistry from *ex vivo* modulation. Many, if not most PAVs, present themselves during collection and initial processing, providing a single window of opportunity to either handle them correctly or not. For example, P/S PAVs such as the type of blood collection tube, proper collection tube filling, number of collection tube inversions, pre-centrifugation delay, and post-centrifugation delay are all completed in a single pass and the record of these PAVs will not ever have an opportunity to change during the life of the P/S specimen. Other PAVs, however, are constantly subject to change.

Of these, the PAV that is arguably the most difficult to control and track over the life of an archived specimen is exposure to thawed conditions. P/S does not fully freeze until the temperature is −30 °C or colder (14, 15). When thawed, a wide range of *ex vivo* biochemical reactions can take place, distorting the portrait of *in vivo* biochemistry that the samples are supposed to reflect. While many if not most researchers who handle P/S know that it should be stored at −80 °C or colder, the facts that not all labs possess a −80 °C freezer, −20 °C freezers are common, P/S appears (visually) to be frozen at −20 °C, and many clinical research protocols allow at least temporary storage of newly collected specimens to be stored at −20 °C for days to weeks before transfer to a −80 °C freezer means that many P/S samples become compromised by temporary storage at −20 °C. Moreover, even after placement in a −80 °C freezer, an ever-present risk of exposure to thawed conditions remains in place for the life of every aliquot of P/S. For example, −80 °C freezer failures occur, delays during sample shipments may result in the loss of dry ice and induce thawing, and there may be need(s) to re-aliquot samples. Thus, thawing remains a constant threat to the integrity of all P/S samples until they have been analyzed.

**Fig. 1:**
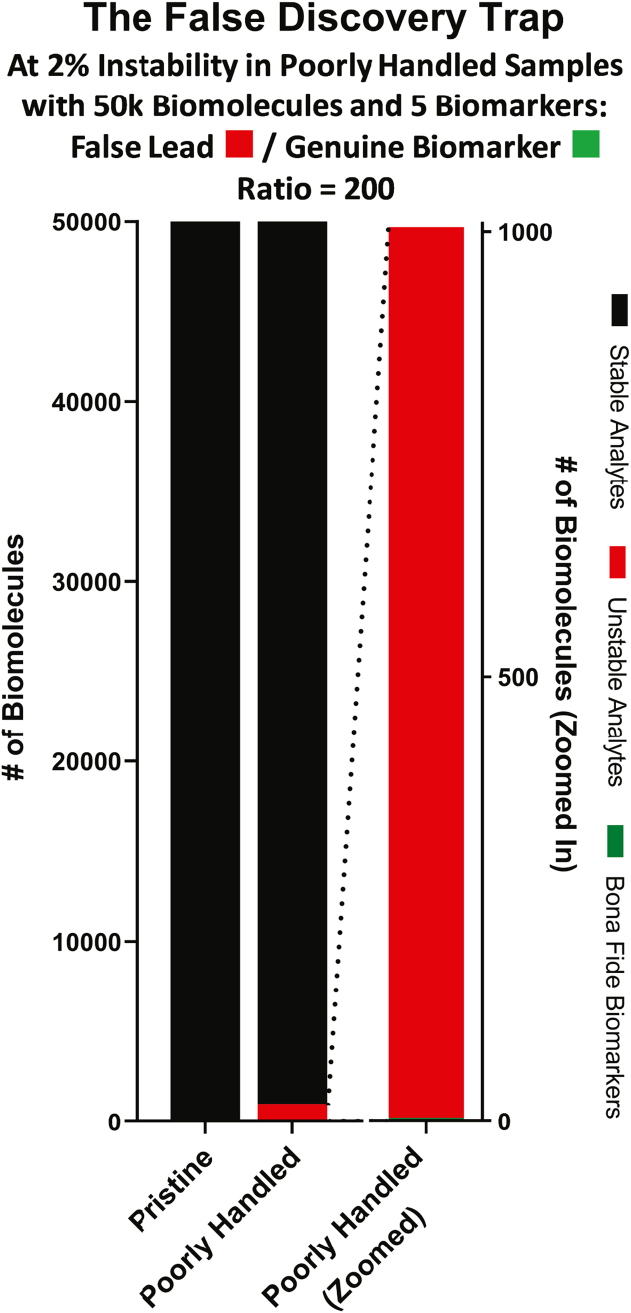
**The False Discovery Trap.** Unstable biomolecules within a poorly handled cohort will change relative to any other cohort with which they are compared, giving them the appearance of biomarkers. Under modest cohort mishandling that introduces quantitative instability into just 2% of analytes, a severe false lead-to-genuine biomarker ratio is introduced, creating a minefield of false discoveries that can be difficult if not impossible to avoid.

For any given collection of samples, the scale of the problem depends on the degree to which specimen biomolecules no longer reflect *in vivo* reality. But it is not immediately obvious that for common biomarker discovery and related investigations, *even a very low percentage of molecules deviating from in vivo reality* presents a major *false discovery trap*: several studies over the past decade have estimated the percentages of different types of biomolecules that are quantitatively altered upon P/S exposures to common thawed conditions (16, 17, 18, 19, 20, 21). They have found that generally low percentages (*i.e.*, 2–20%) of biomolecules are unstable when the specimens in which they reside are exposed to common thawed conditions. On its surface, this gives the appearance that the problem is insignificant. A quick thought experiment, however, demonstrates the opposite: imagine a set of serum samples from stage I lung cancer patients that have been paired with age/gender/smoking-matched samples from at-risk but cancer-free donors to comprise a case/control study. If each sample contains 50,000 different measurable biomolecules (*i.e.*, 50,000 qualitatively different metabolites, proteins, miRNAs, etc.), we might optimistically estimate that five of these might serve as effective markers of stage I lung cancer. If, however, a portion of the case specimens were exposed to a > −30 °C thaw event while the control samples were not (or vice versa—a rather common scenario as we have shown (22, 23)), resulting in statistically significant shifts in the concentration of *only 2%* of exposed-sample biomolecules, this would introduce a *200-fold excess* ((50,000 ∗ 0.02)/5 = 200) of falsely altered biomolecules relative to the number of bona fide biomarkers waiting to be discovered. Hence the *false discovery trap*: the seemingly minor thaw event has introduced a clinical reality–hiding minefield of false/irreproducible “discoveries” waiting to mislead investigators and waste time and money in the process (Fig. 1).

Of course, P/S thawing results in multiple different types of *ex vivo* biomolecular change. Proteins are susceptible to artifactual *ex vivo* posttranslational modifications such as glycation (24, 25, 26), oxidation (both S-cysteinylation (22, 27) and methionine sulfoxidation (27)), and proteolytic degradation (28, 29, 30, 31). Modifications such as these can potentially impact protein quantification without any indication that they have occurred—particularly when quantification is dependent on specific molecular interactions that may be silently disrupted by these modifications (*e.g.*, immunoassays). Numerous examples of apparent changes in protein concentrations occurring due to P/S exposure to thawed conditions are summarized elsewhere (18, 23, 32, 33). Unfortunately, enzymatic, oxidative, and/or other *ex vivo* chemical processes do not solely impact proteins; they impact every major class of biomolecule, including small molecules/metabolites (34, 35, 36), lipids (37, 38), cholesterol (38), peptides (7), nucleic acids such as miRNAs (39), and, to a modest degree, glycans (40, 41, 42). This problem should be of substantial concern to biomedical researchers who employ archived blood P/S in their research, as such evidence suggests that without comprehensive documentation on how these archived P/S samples have been handled and stored, it may be impossible to properly determine their suitability for specific projects.

Unfortunately, documentation alone does not always suffice as sufficient evidence of P/S integrity. We have recently shown *via* two independent incidents that empirical molecular evidence—above and beyond paper trails—may be required to accurately document the integrity of archived P/S samples (22, 23). These discoveries were enabled by our recent development of a biomarker of P/S exposure to thawed conditions known as ΔS-Cys-Albumin (22), which quantifies cumulative exposure of P/S to thawed conditions (*i.e.*, temperatures > −30 °C (14, 15)). In summary, it is a 10-μl, dilute-and-shoot, intact-protein LC/MS-based assay of the relative abundances of albumin proteoforms. The assay is based on the fact that the relative abundance of S-cysteinylated (oxidized) albumin in P/S increases inexorably but to a maximum value under 100% when samples are exposed to temperatures above −30 °C. The difference in the relative abundance of S-cysteinylated albumin (S-Cys-Albumin) before and after an intentional incubation that drives this proteoform to its maximum level is denoted as ΔS-Cys-Albumin. ΔS-Cys-Albumin in fully expired samples is zero. The range (with mean ±95% CI) observed for ΔS-Cys-Albumin in freshly collected plasma is 12 to 29% (20.9 ± 0.75%; n = 97), and in matched serum, it is 10 to 24% (15.5 ± 0.64%; n = 97) (22). Cumulative exposure is calculated *via* a multi-reaction biochemical rate law that we and others have established (22, 43) and that we have shown is applicable to actual plasma and serum samples, enabling back-calculation of the time at which unknown P/S specimens have been exposed to the equivalent of room temperature (22).

Rate law-based linkage of ΔS-Cys-Albumin to the equivalence of exposure time at room temperature provides an intrinsic connection between ΔS-Cys-Albumin and any clinical biomarker with a known stability profile at room temperature. But for candidate clinical biomarkers without known stability profiles or P/S samples that may be known to have only been exposed to refrigeration or −20 °C storage, a definitive connection between ΔS-Cys-Albumin and marker stability remains poorly established. Thus, the purpose of this study was to concurrently evaluate the stability of proteins of interest to pre-clinical research at −20 °C, 4 °C, and 23 °C in conjunction with ΔS-Cys-Albumin measurements in order to begin to forge an empirical linkage between protein stability at all major storage temperatures and ΔS-Cys-Albumin.

## Experimental Procedures

### Experimental Design and Statistical Rationale

The aspects of this study that required substantial planning with regard to methodological design and the logistics of execution included patient enrollment; collection of matched serum, lithium heparin plasma and K_2_EDTA plasma, processing and short-term handling/storage; and execution of thawed-state stability studies at −20 °C, 4 °C, or 23 °C.

The most important details on how these aspects of the study were executed as well as brief descriptions of the analytical procedures are described below. Further detailed information on donor inclusion and exclusion criteria, blood collection and processing protocols, ΔS-Cys-Albumin measurements, and protein measurements is provided in Supplemental Data.

#### Donor Information and Plasma and Serum Specimen Collection

Matched serum, lithium heparin plasma, and K_2_EDTA plasma were collected (in that order) from gastrointestinal (GI) cancer patients and cancer-free donors under informed consent and local IRB approval by the Cooperative Human Tissue Network or Valleywise Health. Specimens were collected in compliance with the Declaration of Helsinki principles. Analysis of the specimens as described in this article was approved by the Arizona State University IRB. Basic donor demographics and disease status information is provided in Table 1. Patients with compromised kidney function (*i.e.*, eGFR <60 ml/min per 1.73 m^2^) were excluded due to the possibility that poor kidney function can, in theory, result in abnormally high ΔS-Cys-Albumin measurements (22).

**Table 1:** Patient characteristics for the baseline ΔS-Cys-Albumin and protein stability studies

Patient demographics	Patient samples for determination of baseline ΔS-Cys-Albumin values	Patient samples for stability study
	n (%)	n (%)
Number of patients	84	24
Sex		
Males	35 (41.7)	13 (54.2)
Females	49 (58.3)	11 (45.8)
Age (years)		
<40	16 (19.1)	3 (12.5)
40–60	44 (52.3)	11 (45.8)
>60	24 (28.6)	10 (41.7)
Ethnicity/race		
White	75 (89.3)	20 (83.3)
Black	7 (8.3)	4 (16.7)
Other	2 (2.4)	0 (0)
Disease status		
Cancer-Free	47 (56.0)	12 (50.0)
Cancer	37 (44.0)	12 (50.0)
Stage I	1 (2.70)	1 (8.3)
Stage III	1 (2.70)	1 (8.3)
Stage III	4 (10.8)	1 (8.3)
Stage IV	10 (27.0)	3 (25.0)
Stage undetermined	21 (56.8)	6 (50.0)

**Fig. 2:**
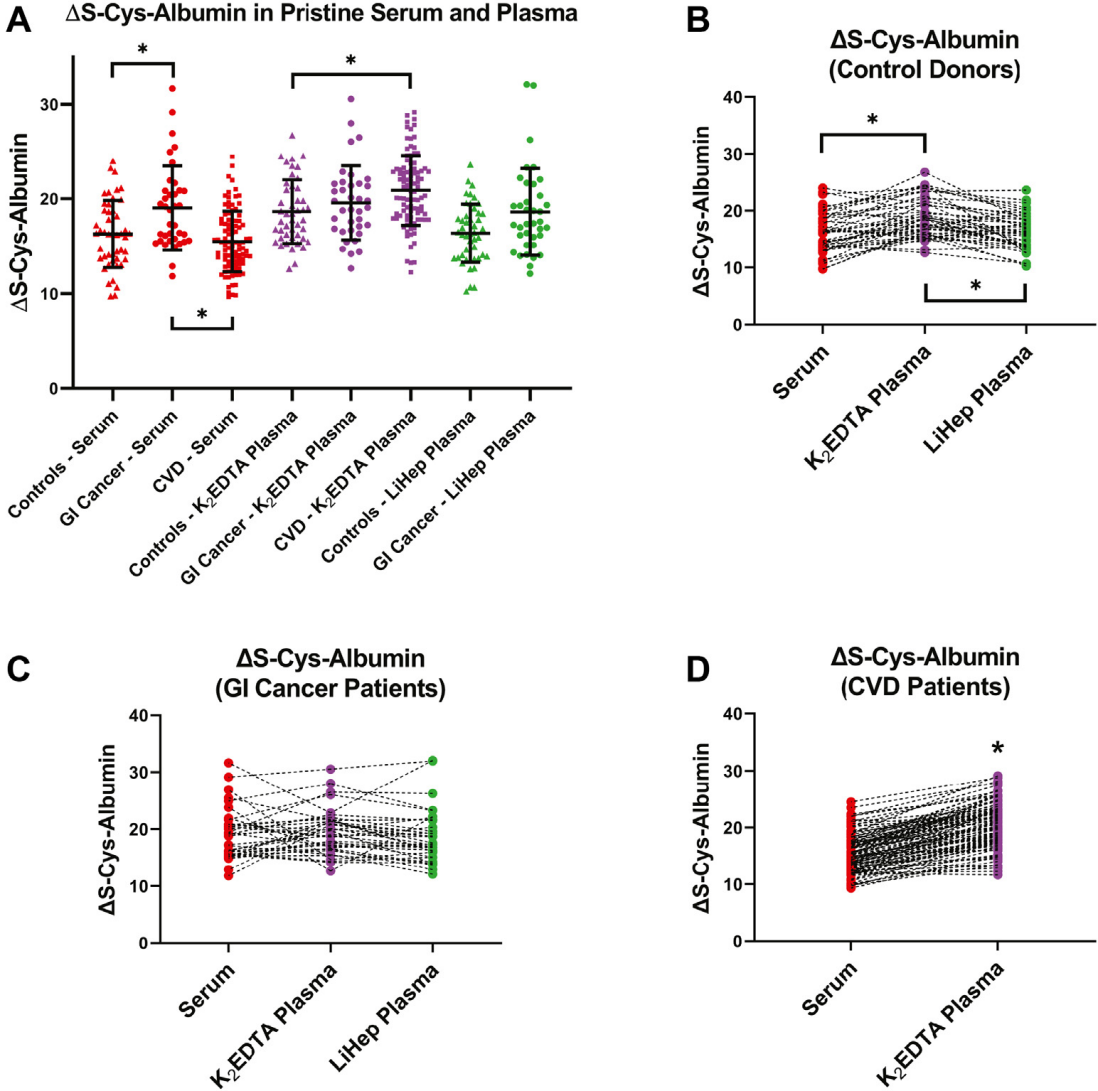
**ΔS-Cys-Albumin in fresh, rapidly processed human serum and matched plasma samples.***A*, comparisons between cancer-free control donors (n = 47), GI cancer patients (n = 37), and non-acute cardiac (CVD) patients (n = 97) for each matrix. Error bars represent mean ± SD; from *left* to *right* these values are: 16.3 ± 3.5, 19.0 ± 4.4, 15.5 ± 3.2, 18.6 ± 3.4, 19.6 ± 3.9, 20.9 ± 3.7, 16.4 ± 3.1, and 18.6 ± 4.6. ∗ indicates a significant difference between means of indicated groups based on a one-way ANOVA with Tukey’s posthoc test or a *t* test. Results were compared to a Bonferroni-corrected *p*-value threshold to account for multiple comparisons (*i.e.*, *p* < 0.05/7 or 0.0071). *B*–*D*, matched collections within each patient group, including (*B*) cancer-free control donors, (*C*) GI cancer patients, and (*D*) CVD patients. ∗ indicates a significant difference between matched sets with *p* < 0.00625; repeated measures (RM) ANOVA with Tukey’s posthoc test or paired *t* test. CVD, cardiovascular disease; GI, gastrointestinal.

In total, 84 patients were enrolled in this study. Thirty seven were GI cancer patients and 47 were cancer-free control donors. ΔS-Cys-Albumin in their matched K_2_EDTA plasma, lithium heparin plasma, and serum was measured at baseline. Of these patients, matched K_2_EDTA plasma and serum from 24 of them were employed for the ΔS-Cys-Albumin time courses. K_2_EDTA plasma from these 24 patients was employed for measurements of the clinically relevant proteins. The data shown in Figure 2 from 97 non-acute cardiac patients were originally described elsewhere (22) and were simply included in Figure 2 for the sake of comparison.

A strict blood collection and processing protocol was followed that included the following: plasma tubes were pre-chilled to 0 to 4 °C. Collection tubes were properly filled (any partially filled tubes were rejected). Immediately after collection, serum tubes were inverted (never shaken) five times and plasma tubes were inverted eight times. Serum was allowed to clot at room temperature for 45 min. Matched plasma was placed on ice while serum clotted, then all tubes were centrifuged at 4 °C. Plasma and serum were then immediately aliquoted on ice. Aliquots were placed in a −80 °C freezer within 2 h from the time of initial draw. To verify the timing of all processing steps, time stamps were recorded at (1) the time of initial draw, (2) time of centrifugation completion, and (3) the time at which aliquots were placed at −80 °C. P/S with a visually estimated degree of hemolysis >250 mg/dl were excluded.

Plasma and serum samples were shipped to Arizona State University overnight on dry ice. Upon receipt, specimens were verified as frozen and then unpacked into a −80 °C freezer equipped with continuous temperature monitoring. All specimens were allowed to sit in the −80 °C for at least 7 days prior to thawing to ensure that any residual CO_2_ in the headspace had been exchanged for air in order to avoid any CO_2_-induced sample acidification effects (44).

#### Initial Analysis and Thawed-State Stability Studies

All samples were randomized prior to analysis using a random number generator for run order assignment. ΔS-Cys-Albumin in pristine, never-thawed aliquots was measured within a time range of 12 to 20 months after initial specimen collection, during which time specimens were kept continually at −80 °C.

Following collection of baseline ΔS-Cys-Albumin values, K_2_EDTA plasma and matched serum from 12 GI cancer patients and 12 cancer-free control donors were selected for inclusion in thawed-state stability studies based on their initial ΔS-Cys-Albumin values being equally distributed across the entire range of initial ΔS-Cys-Albumin values measured. Thawed-state stability studies involved incubation of separate aliquots of these plasma and serum samples for up to 65 days at −20 °C, 28 days at 4 °C, or 96 h at 23 °C. ΔS-Cys-Albumin was measured in both plasma and serum; proteins of clinical interest were only measured in K_2_EDTA plasma.

A separate aliquot for each individual nonbaseline thawed-state time point *and* protein assay (either 20 μl for ΔS-Cys-Albumin or 100 μl for protein measurements) was created from a parent aliquot that had never previously been thawed. To create these aliquots, the parent sample was thawed and kept on ice with strict time tracking for a period of 3.4 ± 1.1 (SD) min for the 23 °C-exposed samples, 8.5 ± 2.0 min for the 4 °C-exposed samples, and 14.2 ± 3.0 min for the −20 °C-exposed samples—by which times all temperature exposure time courses were started. Time course aliquots were immediately placed at −80 °C upon completion of their time/temperature exposure period. Once all time courses were completed, time course aliquots were randomized and ΔS-Cys-Albumin or the clinically relevant proteins were measured in them. Clinically relevant proteins at all stability time points (including baseline) were measured as a randomized set of 240 K_2_EDTA plasma samples by multiplexed immunoassay on either the Luminex 100 or 200 platform by MyriadRBM. Each protein was measured once in each sample. Given that patient specimens were collected over a period of about 1 year, clinically relevant proteins were analyzed within a time range of 18 to 30 months after initial specimen collection. With the exception of intentional thawed-state incubation periods, all specimens were kept continually at −80 °C prior to analysis. After all ΔS-Cys-Albumin and clinically relevant protein time course specimens were analyzed, ΔS-Cys-Albumin was once again measured in a residual never-thawed aliquot of each sample in order to verify the long-term stability of ΔS-Cys-Albumin at −80 °C. This occurred one year after the initial ΔS-Cys-Albumin measurements in never-thawed samples were made.

### Statistical Analysis

To evaluate the stability of clinically established protein biomarkers in K_2_EDTA plasma, the concentration measured for each analyte at each condition was compared with its respective control aliquot kept continuously at −80 °C. Data consisted of n = 24 subject samples measured longitudinally over time at temperatures of 23 °C, 4 °C, and −20 °C, respectively. First, we performed descriptive statistics and analyzed ΔS-Cys-Albumin at the baseline data (at time = 0), comparing the effect of health status (cancer *versus* normal), gender (male *versus* female), and race (white *versus* black) using a two-sample *t* test. The age effect was tested using simple linear regression. Second, we analyzed longitudinal ΔS-Cys-Albumin data using linear mixed effects models to account for the repeated measures within subjects. Specifically, we consider the full model as follows:

$$y_{\text{ij}}=\beta_{0}+\beta_{1}{\text{age}}_{i}+\beta_{2}{\text{status}}_{i}+\sum_{j=1}^{q}\beta_{3j}t_{\text{ij}}+\sum_{j=1}^{q}\beta_{4j}{\text{age}}_{i}t_{\text{ij}}+\sum_{j=1}^{q}\beta_{5j}{\text{status}}_{i}t_{\text{ij}}+\varepsilon_{ij}$$

where y_ij_ is the concentration of ΔS-Cys-Albumin for the i^th^ subject at j^th^ time point, age_i_ is the age for the i^th^ subject, status_i_ is 1 if i^th^ subject’s status is cancer and 0 otherwise, t_ij_=1 if y_ij_ is observed at the j^th^ time point, and t_ij_=0 otherwise, ε_ij_ is an error term, and q is the number of time points observed excluding the baseline. We employed a step-down approach that dropped the interaction term of status and time if they were nonsignificant and used reduced models with the main effects only.

The same approach was used to analyze the 21 clinically relevant proteins, with natural logarithm-transformed y values to improve normality. Using the reduced model, the least square means of the y values at each time point were computed and compared between the baseline and any of the other time points using Wald tests. Bonferroni corrections were applied to adjust the calculated *p*-values of the 21 proteins for pairwise comparisons between time points. Bonferroni-adjusted *p*-values less than 0.05 were regarded as statistically significant. All the analyses were conducted at each temperature separately. Serum and K_2_EDTA plasma samples were additionally separated when analyzing ΔS-Cys-Albumin.

Finally, we conducted repeated measures correlation (45) to quantify the association between ΔS-Cys-Albumin and each unstable protein. Pearson correlation coefficients were calculated to quantify the association between ΔS-Cys-Albumin and the percentage of destabilized proteins. We used GraphPad Prism software (version 9.3.1) for descriptive statistics, t-tests, and repeated measures-ANOVA; MIXED procedure in SAS 9.4 (SAS Inst. Inc,) for linear mixed effects models and R 4.1.0 (R foundation, Vienna, Austria); and R rmcorr package for correlation analyses.

### Laboratory Procedures

#### Measurement of ΔS-Cys-Albumin

P/S samples were prepared and the percentage of albumin in the S-cysteinylated form (S-Cys-Albumin) was measured as previously described (22, 27). Briefly, 1 μl of plasma or serum was diluted 1000-fold in 0.1% (v/v) TFA and injected onto an LC-MS instrument where albumin was concentrated and desalted on a protein cap-trap, then eluted directly into the mass spectrometer for measurement of the intact protein and relative quantification of its proteoforms. Nine microliters of the same plasma or serum sample were then placed in a 0.6-ml polypropylene Eppendorf snap-cap tube and incubated in a dry oven at 37 °C for 24 h. One microliter of this sample was then diluted 500-fold in 0.1% TFA and injected onto the LC-MS for analysis. ΔS-Cys-Albumin is defined as the *difference* between the percentage of albumin in the S-cysteinylated form before and after the overnight incubation at 37 °C that drives the percentage of S-Cys-Albumin to its maximum value (22). Details of the LC-MS method, including data processing are provided in Supplemental Data.

#### Measurement of Clinically Relevant Proteins

Twenty-five clinically relevant proteins were measured *via* three multi-analyte profile assays (MAPs) in each 100-μl K_2_EDTA plasma aliquot from the thawed-state stability studies. MAPs were preconfigured and validated by MyriadRBM and included “HCANCER2”, “HMP8”, and “HMPC38”.

The HCANCER2 MAP included amphiregulin, epidermal growth factor (EGF), epidermal growth factor receptor (EGF-R), epiregulin, heparin-binding EGF-like growth factor (HB-EGF), placenta growth factor (PGF), platelet-derived growth factor BB (PDGF-BB), and tenascin-C.

The HMP8 MAP included adiponectin, alpha-2-macroglobulin (A2M), ferritin, myoglobin, plasminogen activator inhibitor-1 (PAI-I), T-cell-specific protein RANTES, tissue inhibitor of metalloproteinases-1 (TIMP-1), tumor necrosis factor receptor-2 (TNF-R2), vascular cell adhesion molecule-1 (VCAM-1), EN-RAGE, and pulmonary and activation-regulated chemokine (PARC).

The HMPC38 MAP included alpha-fetoprotein (AFP), cancer antigen 125 (CA-125), cancer antigen 19-9 (CA-19-9), carcinoembryonic antigen (CEA), human chorionic gonadotropin beta (hCG-b), and neuron-specific enolase (NSE).

Samples were measured in random order by trained MyriadRBM personnel who thawed samples for the minimal required time and kept them on ice when thawed prior to analysis. Comprehensive assay validation data are available from MyriadRBM or the corresponding author upon request.

Four of the above proteins were measured but were below the limit of quantification (LOQ) in the vast majority of samples and were therefore not reported here. These included amphiregulin, epiregulin, PGF, and hCG-b. For a few of the 21 proteins reported on here, one or more patients had concentrations that were at or below the LOQ for all stability time points. These included alpha-fetoprotein (14 patients always below LOQ), CA-19-9 (6 such patients), and CEA (1 such patient). Data on these proteins from such patients were excluded from statistical analysis. There were also instances in which at least one but not all protein concentrations from a given patient were reported as being at or below the LOQ. This occurred at least once for AFP, CA-125, CA-19-9, CEA, EGF, HB-EGF, and PDGF-BB. (supplemental Fig. S3 provides a graphical view of the overall minor extent of these occurrences.) These data points were included in the statistical analysis as being at the LOQ because they were known to not be higher than the LOQ and, for baseline measurements (time = 0), this information alone was potentially helpful in facilitating the detection of a protein instability when protein concentrations apparently increased over time—which constituted the vast majority of instabilities detected (such as that of EGF). In all such cases, had the LOQ been lower, it would have enhanced rather than diminished the magnitude of an instability finding. Consistency in this practice, however, meant that including nonbaseline measurements at the LOQ tended to otherwise blunt the ability to statistically detect unstable proteins.

## Results

### ΔS-Cys-Albumin Baseline Values in Fresh Plasma and Serum Samples

In 2019, we published baseline values for fresh, rapidly processed, matched K_2_EDTA plasma and serum samples from 97 non-acute patients with cardiovascular disease (CVD) (22). The clinical characteristics of these donors with CVD are described elsewhere (22) but their baseline ΔS-Cys-Albumin data are provided here for comparison alongside new data from 37 GI cancer patients and 47 cancer-free control donors. (Illustrative raw and charge deconvoluted mass spectral data are provided elsewhere (27).) Matched LiHep plasma was also collected from the GI cancer patients and cancer-free control donors (Fig. 2). An elevation of ΔS-Cys-Albumin in the serum of GI cancer patients compared to the other two patient cohorts was observed. Similarly, ΔS-Cys-Albumin in K_2_EDTA plasma was higher in CVD patients than in the cancer-free control donors. The difference in ΔS-Cys-Albumin between LiHep plasma groups did not quite reach statistical significance (Fig. 2*A*). Within CVD patients and cancer-free controls, ΔS-Cys-Albumin in K_2_EDTA plasma was higher than that of serum in matched collections; and in the cancer-free controls, it was also higher than LiHep plasma (Fig. 2, *B*–*D*). No differences in matched collections were observed, however, between the three matrices in GI cancer patients. Baseline ΔS-Cys-Albumin values in chemotherapy patients (not annotated in Fig. 2) did not differ from cancer patients who were not on chemotherapy at the time of blood draw (*t* test; *p* > 0.5). The range (with mean ±95% CI) observed for ΔS-Cys-Albumin in fresh (baseline) GI cancer patient serum was 12 to 32% (19.0% ± 1.5%). In their K_2_EDTA plasma, it was 13 to 31% (19.6% ± 1.3%). And in their lithium heparin plasma, it was 12 to 32% (18.6% ± 1.5%). For the cancer-free controls, these values for serum were 10 to 24% (16.3% ± 1.0%), for K_2_EDTA plasma were 13 to 27% (18.6% ± 1.0%), and for lithium heparin plasma were 10 to 24% (16.4% ± 0.92%). For comparison and as described elsewhere (22), non-acute CVD patient serum was 10 to 24% (15.5% ± 0.64%) and their K_2_EDTA plasma was 12 to 29% (20.9% ± 0.75%).

Effects of age, gender, and race on baseline ΔS-Cys-Albumin values in the GI cancer patients and cancer-free control donors were also assessed. No significant differences were observed between genders or races for any matrix (t-tests; *p* > 0.05; see Table 1 for n-values). ΔS-Cys-Albumin was slightly but consistently linearly correlated with age in all three matrices (supplemental Fig. S1). In serum, slope = 0.079, ΔS-Cys-Albumin units/yr, *p* = 0.015, Pearson correlation coefficient (r) = 0.26; in K_2_EDTA plasma, slope = 0.074 ΔS-Cys-Albumin units/yr, *p* = 0.013, r = 0.27; and in LiHep plasma, slope = 0.064 ΔS-Cys-Albumin units/yr, *p* = 0.039, r = 0.23. After adjusting for age, the differences between GI cancer patients and cancer-free controls for serum and LiHep plasma (Fig. 2*A*) were diminished but not eliminated (*t* test; serum, *p* = 0.020; K_2_EDTA plasma, *p* = 0.63; LiHep plasma, *p* = 0.029). And as expected, adjustment for age did not alter the fact that ΔS-Cys-Albumin was significantly higher in K_2_EDTA plasma relative to serum and LiHep plasma in matched collections from the cancer-free control patients.

### ΔS-Cys-Albumin Time Courses at 23 °C, 4 °C, and −20 °C

A separate aliquot for each individual time point was created from parent samples that had never previously been thawed. To create these aliquots, the parent sample was thawed and kept on ice for strictly limited and documented time periods as described in the Experimental Procedures section. ΔS-Cys-Albumin was measured at 9 to 11 different time points per exposure temperature (23 °C, 4 °C, or −20 °C) in K_2_EDTA plasma and serum aliquots from 12 GI cancer patients and 12 cancer-free donors.

**Fig. 3:**
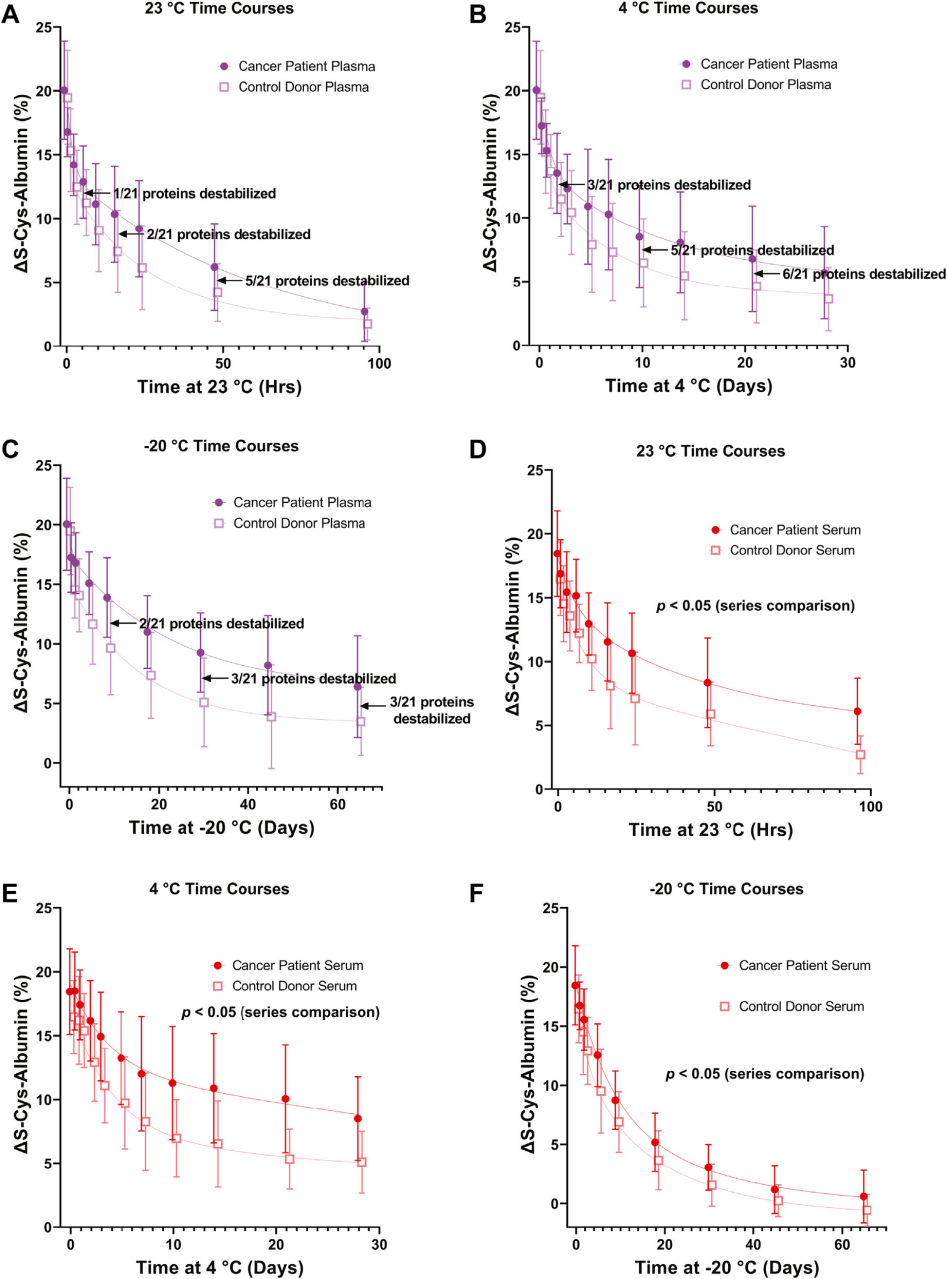
**ΔS-Cys-Albumin time courses at 23 °C, 4 °C, and −20 °C.** ΔS-Cys-Albumin profiles over time in K_2_EDTA plasma at (*A*) 23 °C, (*B*) 4 °C, and (*C*) −20 °C, and in serum at (*D*) 23 °C, (*E*) 4 °C, and (*F*) −20 °C. Jitter was added to the x-axis to prevent data point/error bar overlap. Mixed effects models indicated that the differences between GI cancer patients and cancer-free control donors were not statistically significant in K_2_EDTA plasma time courses but were significant in serum time courses at all three temperatures (Bonferroni-adjusted *p* < 0.05). Modeled interactions of patient health status × time and patient age × time were not statistically significant in any matrix or at any temperature (Bonferroni-adjusted *p* < 0.05) — meaning that there was no statistical evidence that patient age or health status impacted the manner in which ΔS-Cys-Albumin decayed over time. n = 12 patient samples per data point. Error bars indicate SD. Lines are fitted 2-phase exponential decay curves that are intended to serve only as visual guides. GI, gastrointestinal.

K_2_EDTA plasma and serum results are shown separately (Fig. 3) because, as mentioned above and as we have previously described, K_2_EDTA plasma and serum both tend to have different initial ΔS-Cys-Albumin values in matched samples and different rates of change when the samples are exposed to thawed conditions (22). Control measurements of ΔS-Cys-Albumin in never-thawed aliquots of these K_2_EDTA plasma and serum samples that were kept continuously at −80 °C for approximately 1 year after the initial baseline ΔS-Cys-Albumin measurements were made showed no significant change from their original measurements (supplemental Fig. S2). An initial mixed effects model confirmed a strong interaction between matrix type and time (*p* < 0.001 at all temperatures). As such, K_2_EDTA plasma and serum time courses were subsequently analyzed separately by mixed effect models that included patient age, health status, and time at the indicated temperature as main effects and patient age × time and health status × time as interactions.

Neither interaction was found to be statistically significant in either K_2_EDTA plasma or serum—meaning that there was no statistical evidence that patient age or health status impacted the manner in which ΔS-Cys-Albumin decayed over time. Data were re-analyzed after eliminating these interactions from the model in order to robustly identify significant main effects: as expected, exposure time at all three temperatures resulted in strongly significant changes in ΔS-Cys-Albumin values (Bonferroni-adjusted *p* < 1 × 10^−8^). Patient health status did not significantly impact overall K_2_EDTA plasma time course results at any temperature (Fig. 3, *A*–*C*), but it did significantly impact overall serum time course results at all three temperatures (Bonferroni-adjusted *p* < 0.05; Fig. 3, *D*–*F*). This indicated that GI cancer patient serum samples tended to have slight to modestly higher ΔS-Cys-Albumin values than the cancer-free control donors regardless of any thawed-state exposure that may have occurred. Overall, these results are consistent with the fact that ΔS-Cys-Albumin in the serum of cancer patients started (at baseline) at significantly higher values than in the serum of cancer-free donors. (Fig. 2*A*). The mixed effects model also revealed that patient age had a slight but statistically significant impact on overall K_2_EDTA plasma time course results at 4 °C and −20 °C and significantly impacted overall serum time course results at 23 °C (Bonferroni-adjusted *p* < 0.05; time courses stratified by age are not shown). These observations were consistent with the weak correlations of ΔS-Cys-Albumin with patient age observed in fresh K_2_EDTA plasma and serum samples. As noted below, however, the vast majority of clinically relevant proteins in fresh K_2_EDTA plasma samples had much stronger correlations with patient age than did ΔS-Cys-Albumin.

### Clinically Relevant Protein Time Courses at 23 °C, 4 °C, and −20 °C

Twenty-one clinically relevant proteins (supplemental Table S1 and listed above) were measured by Luminex assay in the K_2_EDTA plasma samples from both the GI cancer patients and cancer-free controls at baseline plus three additional time points per temperature (supplemental Fig. S3). At baseline, there were no significant differences in any clinically relevant protein based on gender, race, or health status after correcting for multiple comparisons. Notably, however, all of these proteins except α-fetoprotein (AFP) and carcinoembryonic antigen (CEA) were strongly significantly correlated with patient/donor age (supplemental Table S2).

**Fig. 4:**
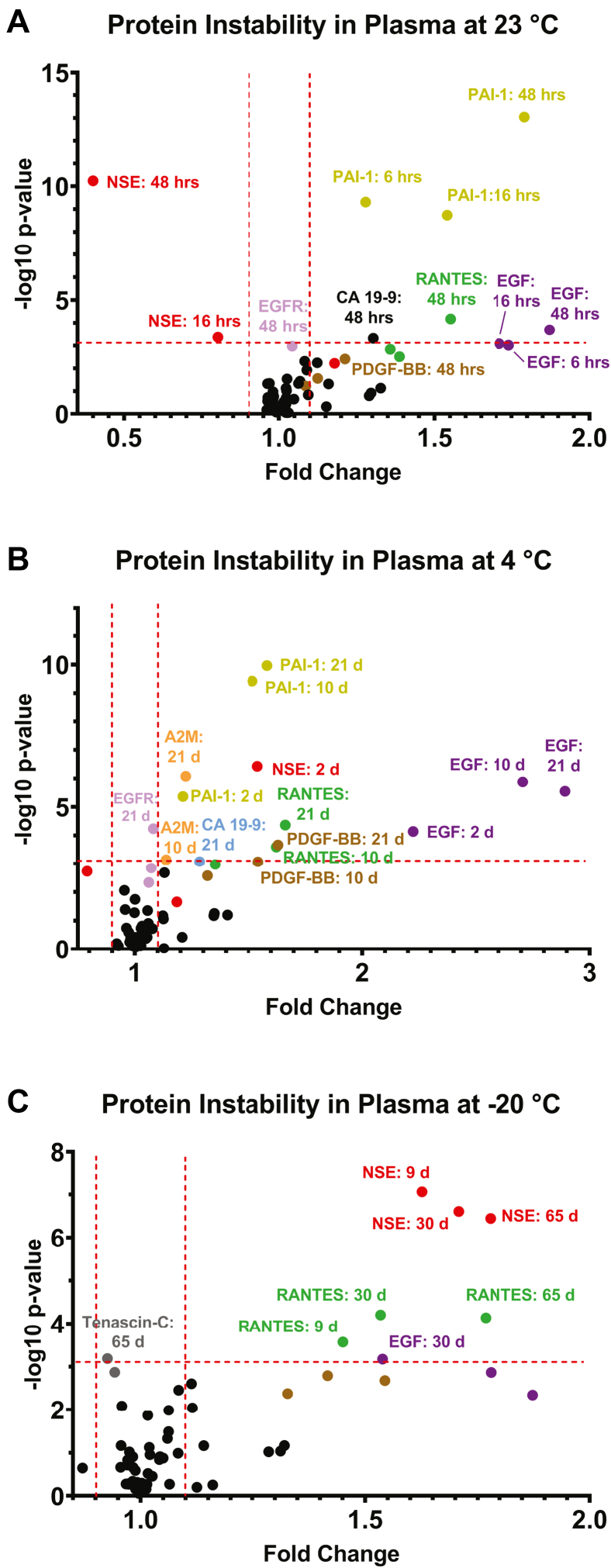
**Clinically relevant protein instability in K**_**2**_**EDTA plasma exposed to thawed conditions.** Volcano plots are shown for (*A*) 23 °C, (*B*) 4 °C, and (*C*) −20 °C. Raw *p*-values were determined using repeated measures–based mixed effects models on natural log-transformed data. Each data point represents the -log_10_*p*-value and mean of measurements made on 24 separate donor samples (including 12 from GI cancer patients and 12 from cancer-free control donors). Proteins in the *upper left* and *upper right* regions delineated by *dashed red lines* representing a −log_10_*p*-value of −log_10_ (0.05/63) = 3.10, and fold change >10% were considered significantly altered by the indicated exposure conditions. GI, gastrointestinal.

Several proteins exhibited instability in K_2_EDTA plasma over time at 23 °C, 4 °C, and −20 °C (Figs. 4 and S3). Repeated measures-based mixed effects models were used to determine which proteins changed in a statistically significant manner over time at each temperature. Interactions of patient age × exposure time and patient health status × exposure time at each temperature were considered (along with patient age, health status, and exposure time as main effects) but no interactions were found to be statistically significant. As such, the mixed effects model was simplified to include only patient age, health status, and exposure time at each temperature as main effects. Following the identification of significantly altered proteins, Wald tests were applied to identify which time point(s) were significantly different from baseline. Here, criteria of raw *p*-value less than 0.05/63 (due there being 21 proteins compared at 3 time points per temperature) or 7.9 × 10^−4^ *and* fold change >10% were both required to consider a protein as significantly altered by a given time-temperature exposure (Fig. 4). The proteins most strongly and consistently impacted by exposure of K_2_EDTA plasma to 23 °C, 4 °C, or −20 °C were neuron-specific enolase (NSE), epidermal growth factor (EGF), plasminogen activator inhibitor-1 (PAI-1), and RANTES (also known as CCL5).

### Relationships Between ΔS-Cys-Albumin and Unstable Clinically Relevant Proteins

**Fig. 5:**
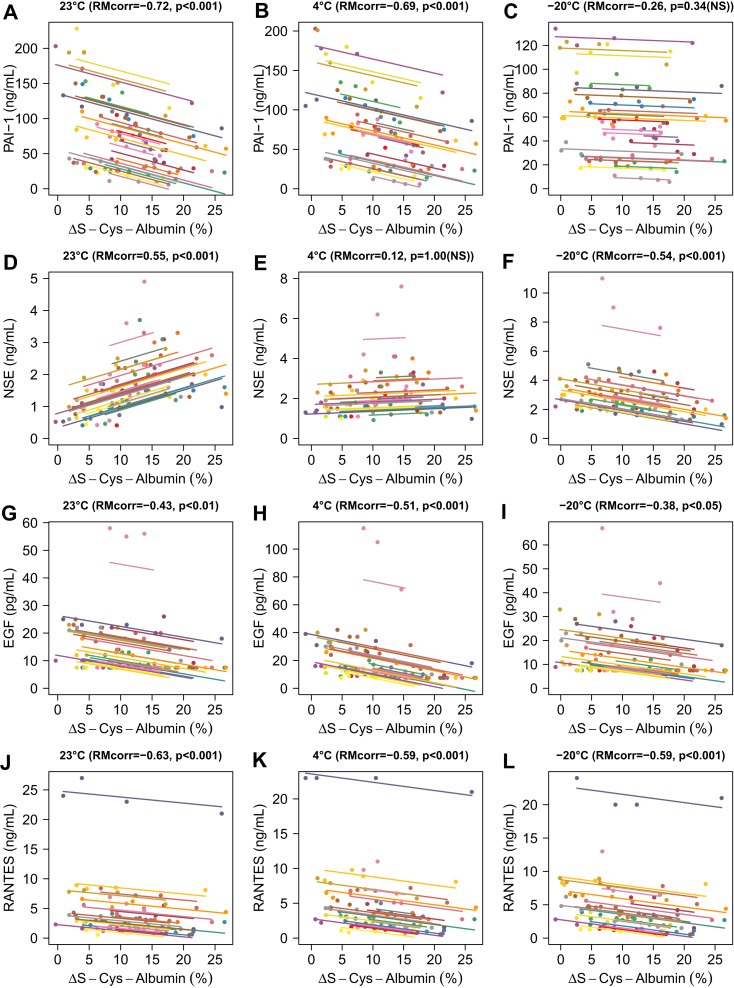
**Relationships between ΔS-Cys-Albumin and specific destabilized proteins.** Repeated measures (RM) correlation plots for plasminogen activator inhibitor-1 (PAI-1) (*A*–*C*), neuron-specific enolase (NSE) (*D*–*F*), epidermal growth factor (EGF) (*G*–*I*), and RANTES (*J*–*L*). The threshold for statistical significance was placed at *p* < 0.05/12 or 0.0042 due to multiple comparisons.

Concurrent quantitative changes in ΔS-Cys-Albumin and each of these four unstable proteins were analyzed for consistency by repeated measures correlation (45). Strong correlations were found at most temperatures (*p* < 0.001, with *p* < 0.05/12 or 0.0042 considered significant due to multiple comparisons) (Fig. 5). EGF, PAI-1, and RANTES all exhibited apparent increases in concentration as ΔS-Cys-Albumin decreased (*i.e.*, with increased exposure time) at all three temperatures. NSE was unique in that at 23 °C and 4 °C, it initially increased but then decreased at longer exposure times (Figs. 5 and S3). At −20 °C, however, it only exhibited an apparent increase over the time span monitored.

**Fig. 6:**
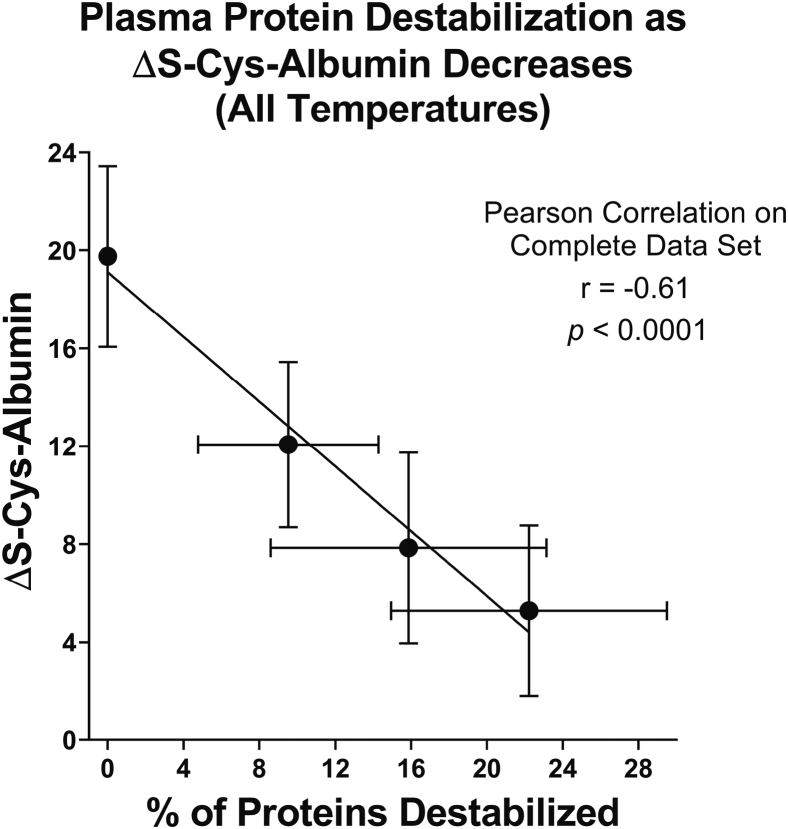
**Relationship between ΔS-Cys-Albumin and the percentage of proteins destabilized in K**_**2**_**EDTA plasma regardless of specific time-temperature exposure.** Each point represents the mean ΔS-Cys-Albumin value at the 0^th^ (n = 24), first (n = 72), second (n = 72), and third (n = 72) exposure time points from all three temperatures (23 °C, 4 °C, and −20 °C) combined. Error bars represent SD. Pearson correlation results shown (r = −0.61, *p* < 0.0001) were based on all 240 individual data points, not just the four mean values shown.

Finally, the relationship between ΔS-Cys-Albumin and the total percentage of destabilized proteins in K_2_EDTA plasma was evaluated, considering the data from all three temperatures together (Fig. 6). Proteins were considered significantly changed based on the *p*-value and fold-change criteria described above for the data in Figure 4. In addition, to account for the increase-then-decrease behavior of NSE (or similarly behaving proteins), once a protein reached the threshold for significant change (destabilization), all future time points for the protein at that temperature were also considered points at which the protein was destabilized. A clear inverse linear relationship was found (r = −0.61 and *p* < 0.0001).

For the 23 °C time course, the once-changed-then-always-subsequently-changed policy noted above pertaining to Figure 6 had no effect. For the 4 °C time course, the policy kept only NSE significant when it might not otherwise have been. But this is appropriate because based on the 23 °C time course (Figs. 4 and S3), as NSE had an excursion in the positive direction then proceeded downward, transitioning through a false negative range. No other proteins in the 4 °C time course were affected by this policy. For the −20 °C time course, the policy kept only EGF at 65 days positive. Without the policy, EGF at 65 days would not have been considered statistically significant within Figure 6 because it had an elevated *p*-value (though its fold-change was actually increased to 1.9-fold at this time point). If the EGF point at 65 days was not considered significant, this would shift the mean data point in Figure 6 that is lowest and furthest to the right on its x-axis to about 20.6%, which would actually bring it closer to the regression line—a result that shows that as it relates to proteins other than NSE, this policy actually caused a slight departure from the linear correlation shown in Figure 6.

## Discussion

ΔS-Cys-Albumin was reported by our group as an endogenous marker of blood plasma and serum exposure to thawed conditions (>−30 °C) in 2019 (22). It is unique among candidate markers for this purpose because it has been extensively characterized and validated: its mechanism of formation is understood; the multi-reaction rate law that governs albumin S-cysteinylation at 23 °C was established and can be used to approximate exposure times of unknown samples; the population reference range for fresh samples from CVD patients was determined in 2019 (and has now been estimated here for GI cancer patients and cancer-free controls); it has passed both group-wise and individual sample-level blind challenges; and it has been employed with “real life” samples to detect previously undisclosed thawed-state exposures of nominally pristine samples being employed for biomarker discovery and validation purposes (22, 23). The 2019 paper (22) provides the information needed for laboratories to set guidelines on how ΔS-Cys-Albumin values in unknown samples can be used to evaluate the quality of P/S samples prior to starting a proteomic analysis. Here, we have extended the potential utility of ΔS-Cys-Albumin by generating empirical stability time course profiles from two dozen individual patients for both serum and K_2_EDTA plasma at −20 °C, 4 °C, and 23 °C. Moreover, we have empirically linked ΔS-Cys-Albumin to the stability of 21 clinically relevant proteins (as measured by Luminex assay) and have shown how drops in plasma ΔS-Cys-Albumin below the range observed in pristine samples can serve as a surrogate indicator to estimate the percentage of immunoassay-measured proteins that have been destabilized in mishandled specimens (Fig. 6).

Such empirical linkage of ΔS-Cys-Albumin to protein stability is useful for situations in which the stability of (pre)clinically important protein(s) of interest at room temperature is not known and therefore cannot be linked to a ΔS-Cys-Albumin cutoff threshold *via* its established rate law. This linkage, combined with the established mechanism of instability, known rate law, and other validation criteria described above make ΔS-Cys-Albumin the most thoroughly characterized and validated marker of blood P/S exposure to thawed conditions to date—by a substantial margin.

In this study, we extended measurements of ΔS-Cys-Albumin in freshly collected and processed (baseline) samples from matched serum and K_2_EDTA plasma to include matched LiHep plasma as well. ΔS-Cys-Albumin in LiHep samples was the same as in serum samples, but ΔS-Cys-Albumin in K_2_EDTA plasma of healthy and CVD patients tended to run a bit higher than matched serum (and LiHep) plasma. ΔS-Cys-Albumin in freshly collected and processed samples from cancer patients, however, was the same across all three matched matrices. The mechanism behind this discrepancy is not clear but seems to be driven by modestly elevated levels of ΔS-Cys-Albumin in cancer patient serum *versus* serum from the other two patient cohorts (Fig. 2*A*). In fact, though it may not have quite reached statistical significance, ΔS-Cys-Albumin was modestly elevated in every matrix from cancer patients relative to the cancer-free control donors.

Elevated ΔS-Cys-Albumin could be caused by high cysteine/cystine concentration and/or low albumin concentrations. The most likely cause of elevated cysteine/cystine is poor renal function (22, 46), but patients with compromised renal function (eGFR <60 ml/min per 1.73 m^2^) were excluded from this study. Hypoalbuminemia, however, is common in cancer patients (47) and can be associated with chemotherapy (48) as well as diminished patient survival rates (49). Most cancer patients were not undergoing chemotherapy at the time of blood collection, and those that were did not have different ΔS-Cys-Albumin values. Absolute concentrations of albumin were not determined, leaving modest hypoalbuminemia as the most likely explanation for slightly elevated ΔS-Cys-Albumin in the cancer patients relative to controls. (Notably, even after correction for age, the significant difference in baseline serum ΔS-Cys-Albumin between cancer patients and cancer-free donors remained.) The source of the difference in ΔS-Cys-Albumin between serum and plasma was investigated in 2019 (22). No definitive explanation was identified, but the pre-centrifugation time difference between plasma and serum as well as serum clotting time were ruled out as contributors to this phenomenon.

The slight increase in baseline ΔS-Cys-Albumin observed with patient age was consistent with known decreases in P/S albumin concentration and modest increases in cysteine/cystine with age (50, 51). Notably, most of the clinically relevant proteins in baseline specimens were more strongly correlated with patient/donor age than was ΔS-Cys-Albumin (supplemental Table S2).

Variability among individual patient time course profiles is expected (22). The results observed here (Fig. 3) are in line with the theoretical ΔS-Cys-Albumin decay curves at 23 °C based on the previously established rate law and known population reference ranges for starting concentrations of reactants and products (22). Serum tends to run at the upper limit predicted, but this probably has to do with the difficulty in estimating the concentration of catalytically available copper in the original model (22). This estimation is difficult because approximately 95% of copper is bound to ceruloplasmin in serum (52) where it is much less catalytically available relative to K_2_EDTA plasma (22) where it has been extracted from ceruloplasmin and is mostly bound to EDTA.

The observation that ΔS-Cys-Albumin in serum decreases at least at fast, if not faster, at −20 °C compared to its rate of decrease at 4 °C was unexpected. Plasma and serum are partially (noneutectically) frozen at −20 °C (14, 15, 53). This creates a non-native system of reactants and products that are likely differently concentrated compared to when all components of plasma or serum are in the liquid state. The rate laws for the relevant reactions have not been determined at 4 °C or −20 °C, but regardless of whether or not the reactions exhibit Arrhenius behavior, it remains possible that this differently concentrated state may lead to a situation in which the reactions run faster despite the colder temperature. Of course, this may also be reflected in the rates of other biochemical reactions—hence the need for an *empirical* linkage between ΔS-Cys-Albumin and proteins of clinical interest.

Additionally, as we have previously observed, copper is less catalytically available in serum than it is in EDTA plasma—which is likely due to the fact that it is mostly bound to ceruloplasmin in serum (22). This may account for the fact that this effect is only observed in serum—that is, in a partially frozen state at −20 °C, ceruloplasmin’s structure may be altered, allowing copper to become abnormally catalytically available. At this point, however, this conjecture is strictly speculative.

The mechanisms behind the documented protein instabilities are only partially understood at this point. PAI-I is known to be released from platelets during freeze-thaw cycles (54) and in the presence of residual thrombin activity (55). For the other proteins that exhibited instability, it is logical to conclude that biomolecular changes that took place upon exposure to thawed conditions resulted in disruption of protein epitope(s) that were involved in mediating the protein interaction-based quantification. The mechanisms behind these disruptions may include artifactual oxidation (especially of free cysteine and/or methionine residues (56, 57, 58), cf. supplemental Table S1), proteolysis, or other forms of *ex vivo* posttranslational modification (24)—even, theoretically, disturbances to tertiary or quaternary protein structure that disrupt epitopes involved the quantification process. Interestingly, NSE has a particularly large number of free cysteine residues (supplemental Table S1) that may be susceptible to *ex vivo* oxidation by mechanisms akin to those that drive albumin S-cysteinylation (22, 27, 59). A list of proteins measured in this study alongside details of the clonality (*i.e.*, polyclonal or monoclonal) of capture and detection antibodies that were employed is provided in supplemental Table S1. While one might expect assays that rely monoclonal antibodies to be more susceptible to disruption in target protein epitopes, no clear pattern emerged from this information that would suggest that the clonality of antibody reagents employed had a major impact on the observed stability of plasma proteins.

## Conclusions

The work presented here reinforces the fact that the exposure of human plasma to thawed-state conditions (*i.e.*, > −30 °C) can lead to apparent quantitative changes in nontrivial percentages of clinically important proteins as measured by molecular interaction–based assays (*e.g.*, antibody-based protein assays) (17, 60, 61, 62, 63). Concurrent measurements of ΔS-Cys-Albumin and the concentrations of 21 proteins of interest to (pre)clinical research provided a direct empirical linkage between these clinically important proteins and ΔS-Cys-Albumin—allowing ΔS-Cys-Albumin to be used as a surrogate indicator of the suitability of plasma samples for analysis of the clinically relevant protein(s). Additionally, the data presented here show how ΔS-Cys-Albumin measurements can approximate the overall extent to which the proteome of plasma sample(s) has been altered (as detected by molecular interaction–based assays) due to exposure to thawed-state conditions. Therefore, ΔS-Cys-Albumin can now be used as *both* a marker of cumulative exposure to thawed-state conditions as well as an estimator of cumulative damage to clinically important proteins present in plasma. This latter feature, facilitated by the data in Figure 6, may serve as an effective means of using ΔS-Cys-Albumin measurements to empirically gauge the risk of false discovery when employing archived specimens for research purposes.

## Data Availability

All data are presented within the article and/or supplemental data in graphical and/or tabular format. More detailed versions of the data, including limited numbers of raw mass spectra are available upon request from the corresponding author. All raw mass spectrometry files have been uploaded to the MassIVE database at https://massive.ucsd.edu/ and made publicly accessible. The data set was assigned the identifier “MSV000090061” with the name “Delta-S-Cys-Albumin Baseline and Time Courses for Stability Linkage to Other Proteins”, https://doi.org/10.25345/C5K35MJ29 (for viewing without downloading), and FTP download link: ftp://massive.ucsd.edu/MSV000090061/. The data set is 1.02 TB in size.

## Supplemental data

This article contains supplemental data. References cited in the supplemental data include reference (22).

## Conflict of interest

The authors declare that they have no conflicts of interest with the contents of this article.
