# Supplementary material for: Tracking the Stability of Clinically Relevant Blood Plasma Proteins with Delta-S-Cys-Albumin—A Dilute-and-Shoot LC/MS-Based Marker of Specimen Exposure to Thawed Conditions
Source: Mol Cell Proteomics. 2022 Sep 28;21(11):100420. doi: 10.1016/j.mcpro.2022.100420 (PMC9637815; doi:10.1016/j.mcpro.2022.100420)
Supplement: Supplemental data [file mmc1.pdf]

Supplemental Data

Tracking the Stability of Clinical Blood Plasma Proteins with ΔS-Cys-Albumin—a Dilute-and-Shoot LC/MS Based Marker of Specimen Exposure to Thawed Conditions

Running Title: Linking ΔS-Cys-Albumin to Clinical Protein Stability

Erandi P. Kapuruge †,[a,b], Nilojan Jehanathan †,[a,b], Stephen P. Rogers [b], Stacy Williams [b], Yunro Chung [b,c], and Chad R. Borges *[a,b]

Supplemental Data Includes

Detailed Methods

Figures S1-S3

Tables S1-S2

Supplemental Data References

DETAILED METHODS

Human Subjects

A total of 84 patients were enrolled in this study, including 37 GI cancer patients and 47 cancer-free control donors. Sixty-five of the 84 patients were enrolled and had samples collected by the NCI-sponsored Cooperative Human Tissues Network (CHTN; Nashville, TN). The remaining 19 patients were enrolled and had samples collected by Valleywise Health (VWH; Phoenix, AZ; a part of the Maricopa county hospital system).

Inclusion and Exclusion Criteria: To qualify for enrollment in this study, GI cancer patients age 18 and older had to present with a malignant gastrointestinal neoplasm of any kind and at any stage. (Patients with other types of cancer such as lung cancer, kidney cancer, skin cancer, etc. were excluded.) GI cancer patients enrolled by CHTN had not yet started chemotherapy when their blood was collected. Eighty eight percent of the GI cancer patients enrolled by VWH were on chemotherapy at the time of blood draw. (Notably, only GI cancer patient samples collected by CHTN were employed in the thawed-state stability (time course) studies.) GI cancer patients were excluded if they presented with chronic kidney disease or any other condition that lowered their eGFR to < 60 mL/min per 1.73m^2.

Cancer-free control donors age 18 and older had to be cancer-free individuals with no history of cancer. Potential cancer-free donors were excluded if they had chronic kidney disease or if, for any reason, their eGFR was < 60 mL/min per 1.73m^2.

Blood Collection and Processing Protocol:

Plasma tubes were pre-chilled to 0-4 °C. Serum tubes were not pre-chilled. Blood was drawn by venipuncture with an 18 or 20 gauge needle into a 10-mL serum vacutainer tube (without separator gel), then a 2-mL lithium heparin plasma vacutainer tube, and finally a 10-mL K_2EDTA plasma vacutainer tube. Collection tubes were properly filled with blood; any partially filled tubes were rejected. Immediately after collection serum tubes were inverted (never shaken) five times and plasma tubes were inverted eight times. Serum was allowed to clot at room temperature for 45 minutes. Matched plasma was placed on ice while serum clotted, then all tubes were centrifuged in a swing bucket rotor for 10-20 minutes at 2,000 x g and 4 °C. Plasma and serum were then immediately aliquoted on ice. Aliquots were placed in a -80 °C freezer within 2 hours from the time of initial draw. To verify the timing of all processing steps, time stamps were recorded at 1) the time of initial draw, 2) time of centrifugation completion, and 3) the time at which aliquots were placed at -80 °C. Plasma or serum with a visually estimated degree of hemolysis > 250 mg/dL were excluded (20). Unless otherwise specified, samples were kept in a -80 °C freezer until analysis.

<u>Measurement of ΔS-Cys-Albumin by LC/MS</u>

Data Acquisition: Liquid chromatography coupled to electrospray ionization mass spectrometry (LC-ESI-MS) was carried out on a Dionex Ultimate 3000 capillary HPLC

connected to a Bruker maXis 4G quadrupole-time-of-flight (Q-TOF) mass spectrometer. P/S samples were diluted 1000-fold in 0.1% TFA for the first %S-Cys-Albumin measurement and 500-fold for the second %S-Cys-Albumin measurement. Injection volumes of these diluted samples depended on the signal-to-noise ratio (S/N) of daily quality control (QC) samples which, like all specimens, had to have a base peak with a minimum S/N of 50 in the charge deconvoluted spectrum. A trap-and-elute form of LC-MS was carried out in which 1-4 μL of a 500 or 1000-fold diluted P/S sample was loaded via a loading pump at 10 μl/min in 80% water containing 0.1% formic acid (Solvent A) / 20% acetonitrile (Solvent B) onto an Optimize Technologies protein captrap configured for bi-directional flow on a 10-port diverter valve. The trap was then rinsed at this solvent composition with the HPLC loading pump at 10 μl/min for 4 minutes. The flow over the captrap was then switched to the micro pump, which was set at a flow rate of 3 μL/min and composition of 65/35 A/B. This composition was held until 4.5 min. From 4.5-4.6 min. the composition was ramped to 55/45 A/B then ramped to 35/65 A/B from 6.5-6.6 min. From 6.6-7.5 min. the composition was ramped to 20/80 A/B then held for 0.5 min. From 8.0-9.6 min. the composition was ramped back to 65/35 A/B and held until the 10-port diverter valve switched back for loading the next injection. This mobile phase gradient provides nearly complete chromatographic separation of albumin and apolipoprotein A-I (whose charge envelopes are also nearly completely separated by *m*/*z*). Following the valve switch at 4 minutes, the captrap eluate was directed to the

mass spectrometer operating in positive ion, TOF-only mode, acquiring spectra in the m/z range of 300 to 3000. ESI settings for the Agilent G1385A capillary microflow nebulizer ion source were as follows: End Plate Offset -500 V, Capillary -4500 V, Nebulizer nitrogen 3 Bar, Dry Gas nitrogen 4.0 L/min at 225 °C. Data were acquired in profile mode with a digitizer sampling rate of 4 GHz. Spectra rate control was by summation at 1 Hz.

Data Processing: As previously described (20), approximately 0.5 minute of recorded spectra were averaged across the chromatographic peak apex of albumin. The electrospray ionization charge-state envelope was deconvoluted with Bruker DataAnalysis v4.2 software to a mass range of 1000 Da on either side of any identified peak. Charge deconvolution settings were established to ensure that the relative peak widths and signal-to-noise ratios of the raw spectra were reproduced in the deconvoluted spectra. Deconvoluted mass spectra were baseline subtracted and all peak heights were calculated, tabulated and exported to a spreadsheet for further analysis. Peak heights were used for quantification as opposed to peak areas because of the lack of baseline resolution for some albumin proteoforms.

<u>Clinical Protein Measurements</u>

In addition to the details provided in the main text, the following instructions were provided to the MyriadRBM project manager who provided assurance that they were followed:

1) To avoid any possibility of carbon dioxide from the dry ice shipment dissolving in the samples when they are thawed (and thereby potentially acidifying them), please unpack the samples into a -80 °C freezer (NOT a -20 °C freezer!) and leave them for at least 7 days before processing. Since the caps don't seal perfectly, this will allow time for the gas in the sample head space to be replaced by air.

2) Since these samples are part of a stability study it is very important that they spend an absolute minimal amount of time on the bench or at any temperature warmer than -80 °C. Try to expose the samples to one hour or less in the thawed state.

3) When the samples are in the thawed state just prior to analysis, they should be kept on ice whenever possible.

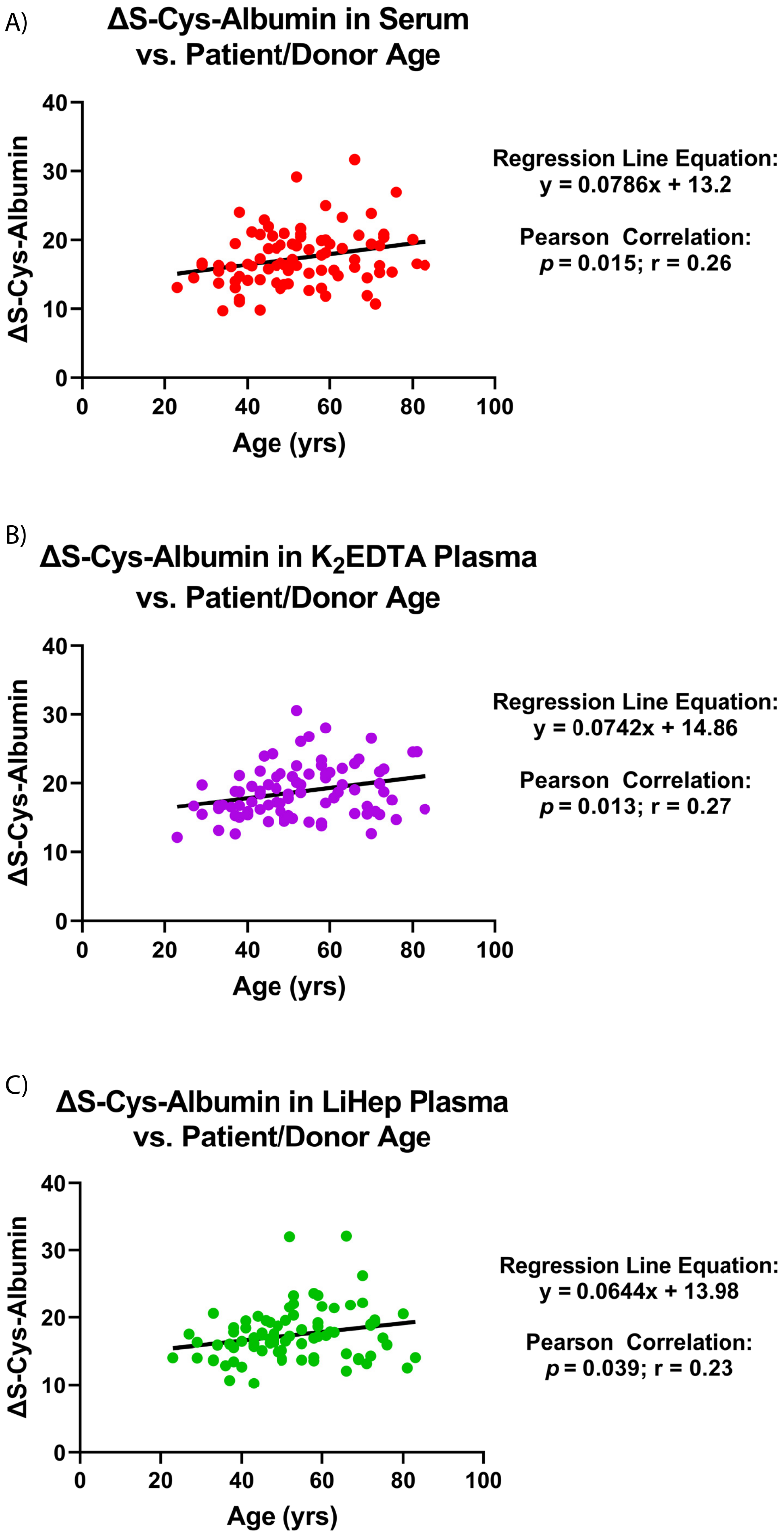
A)
ΔS-Cys-Albumin in Serum
vs. Patient/Donor Age
ΔS-Cys-Albumin
Age (yrs)
Regression Line Equation:
y = 0.0786x + 13.2
Pearson Correlation:
p = 0.015; r = 0.26
B)
ΔS-Cys-Albumin in K2EDTA Plasma
vs. Patient/Donor Age
ΔS-Cys-Albumin
Age (yrs)
Regression Line Equation:
y = 0.0742x + 14.86
Pearson Correlation:
p = 0.013; r = 0.27
C)
ΔS-Cys-Albumin in LiHep Plasma
vs. Patient/Donor Age
ΔS-Cys-Albumin
Age (yrs)
Regression Line Equation:
y = 0.0644x + 13.98
Pearson Correlation:
p = 0.039; r = 0.23

Figure S1

Figure S1: Linear correlations of baseline ΔS-Cys-Albumin with age. In A) Serum, B) K_2EDTA plasma, and C) LiHep plasma.

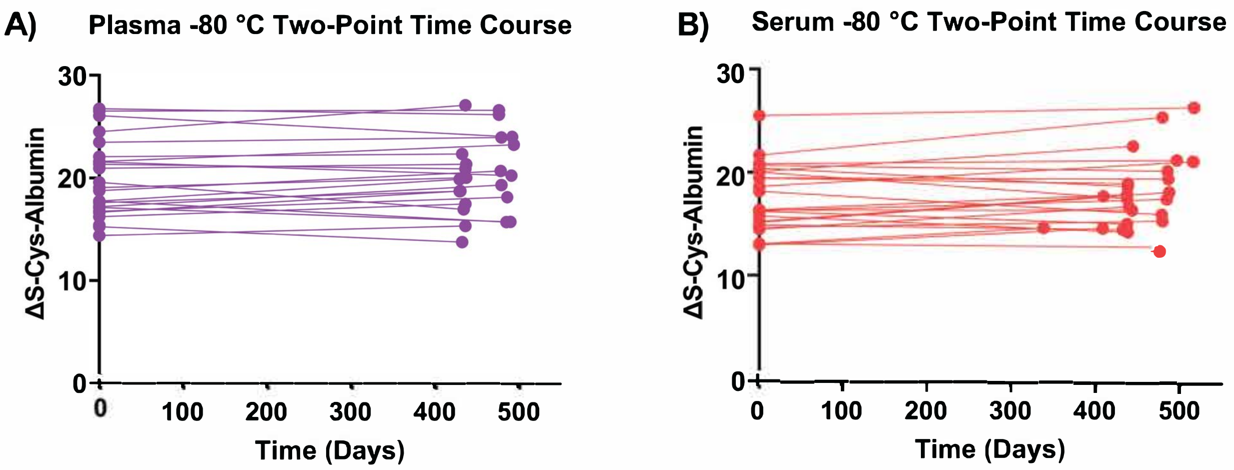
A)
Plasma -80 °C Two-Point Time Course
ΔS-Cys-Albumin
30
20
10
0
0
100
200
300
400
500
Time (Days)
B)
Serum -80 °C Two-Point Time Course
ΔS-Cys-Albumin
30
20
10
0
0
100
200
300
400
500
Time (Days)

Figure S2

Figure S2: Control plasma and serum time courses at -80 °C. Two-point control time courses for A) K_2EDTA plasma and B) serum samples kept at -80 °C for approximately one year. Data were acquired from separate, never-thawed aliquots of the same patient samples from which the time course data in **Fig. 2** were acquired. Time 0 on these plots represents the first day on which each specimen was analyzed. (It was not possible to place the first time point at the actual age of the specimens because in the interest of protecting patient identities the agencies that conducted these prospective collections did not provide the exact dates of collection.) The second data point represents time elapsed since the time 0 data point was acquired. ΔS-Cys-Albumin was not significantly altered in either K_2EDTA plasma or serum ($p > 0.1$; paired t-tests; n = 23 for K_2EDTA plasma (due to loss of one sample) and n = 24 for serum). That stated, the *non-significant* average *relative* percent change for K_2EDTA plasma was +1.8% and for serum was +3.1%.

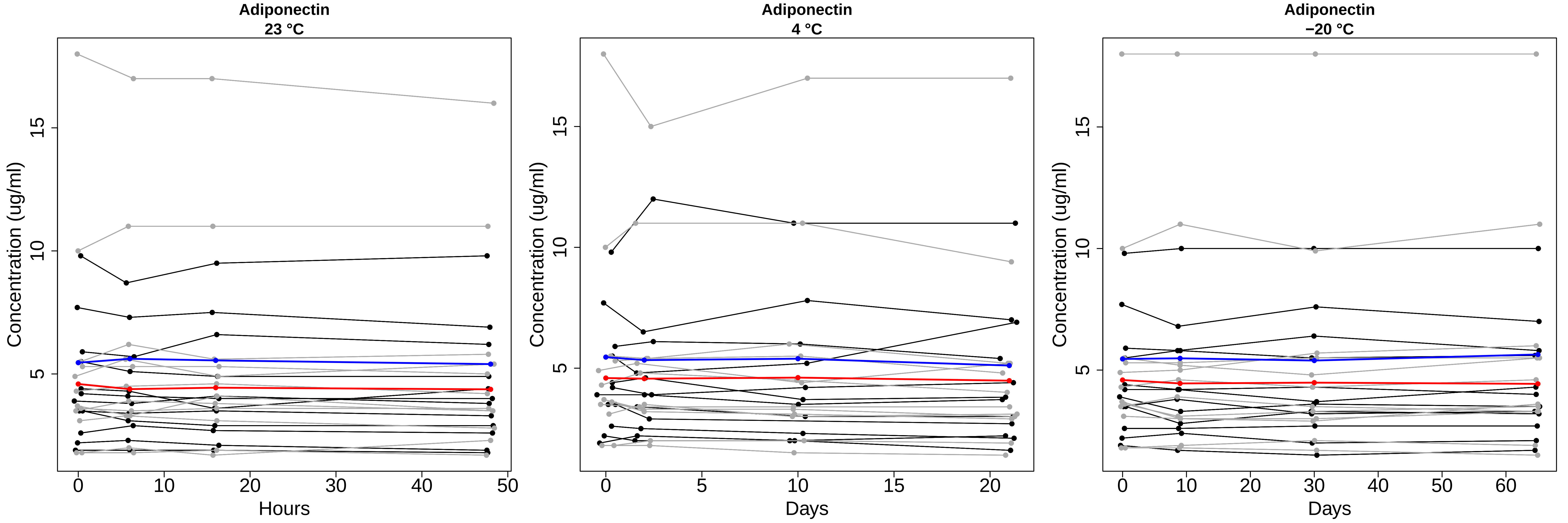

Adiponectin
23 °C
Concentration (ug/ml)
Hours
Adiponectin
4 °C
Days
Adiponectin
−20 °C
Days

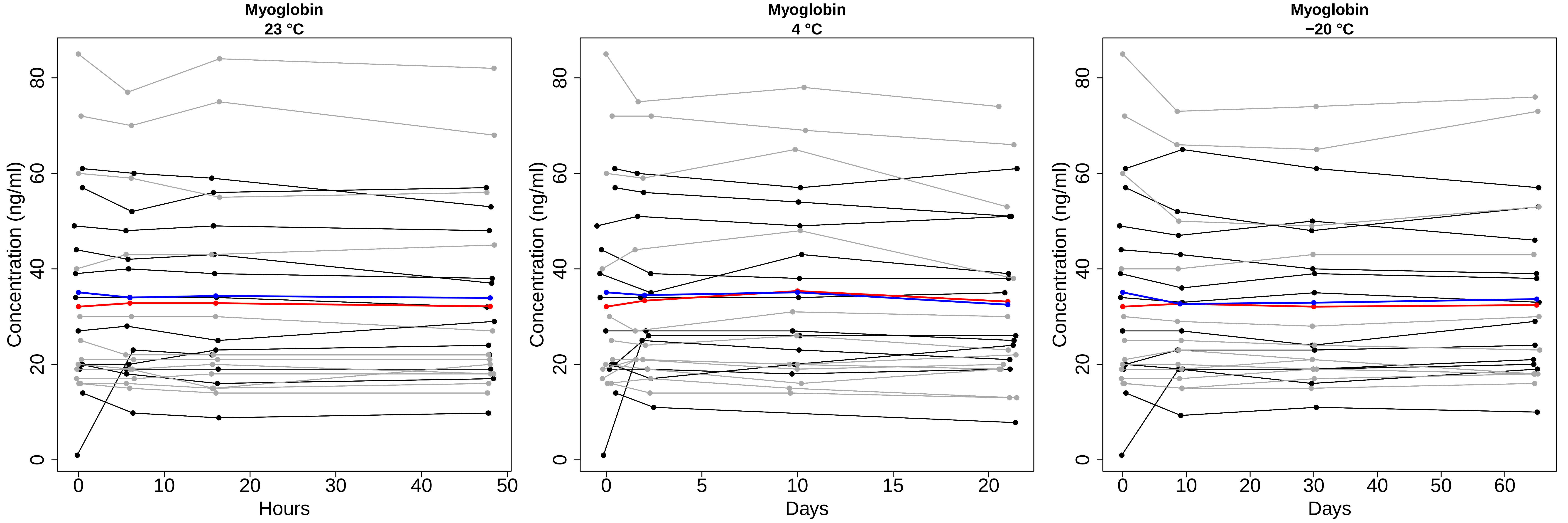
Myoglobin
23 °C
Myoglobin
4 °C
Myoglobin
−20 °C
Concentration (ng/ml)
Hours
Days
Days
0
20
40
60
80
0
10
20
30
40
50
0
5
10
15
20
0
10
20
30
40
50
60

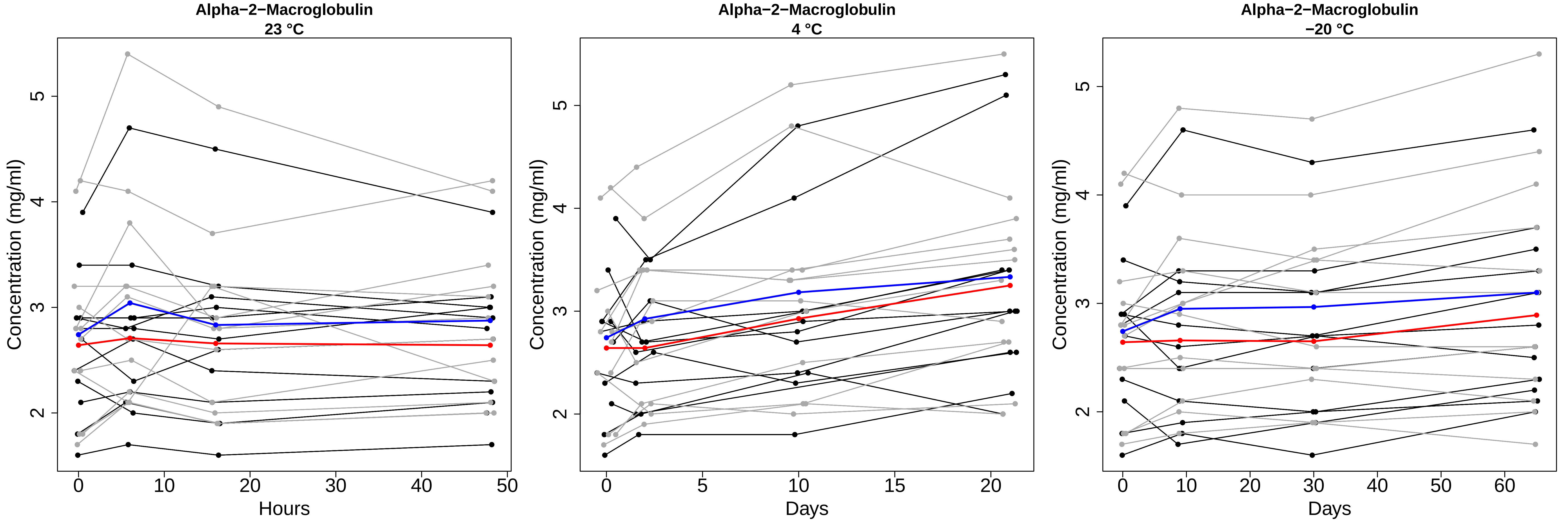
Alpha−2−Macroglobulin
23 °C
Concentration (mg/ml)
Hours
2
3
4
5
0
10
20
30
40
50
Alpha−2−Macroglobulin
4 °C
Concentration (mg/ml)
Days
0
5
10
15
20
Alpha−2−Macroglobulin
−20 °C
Concentration (mg/ml)
Days
0
10
20
30
40
50
60

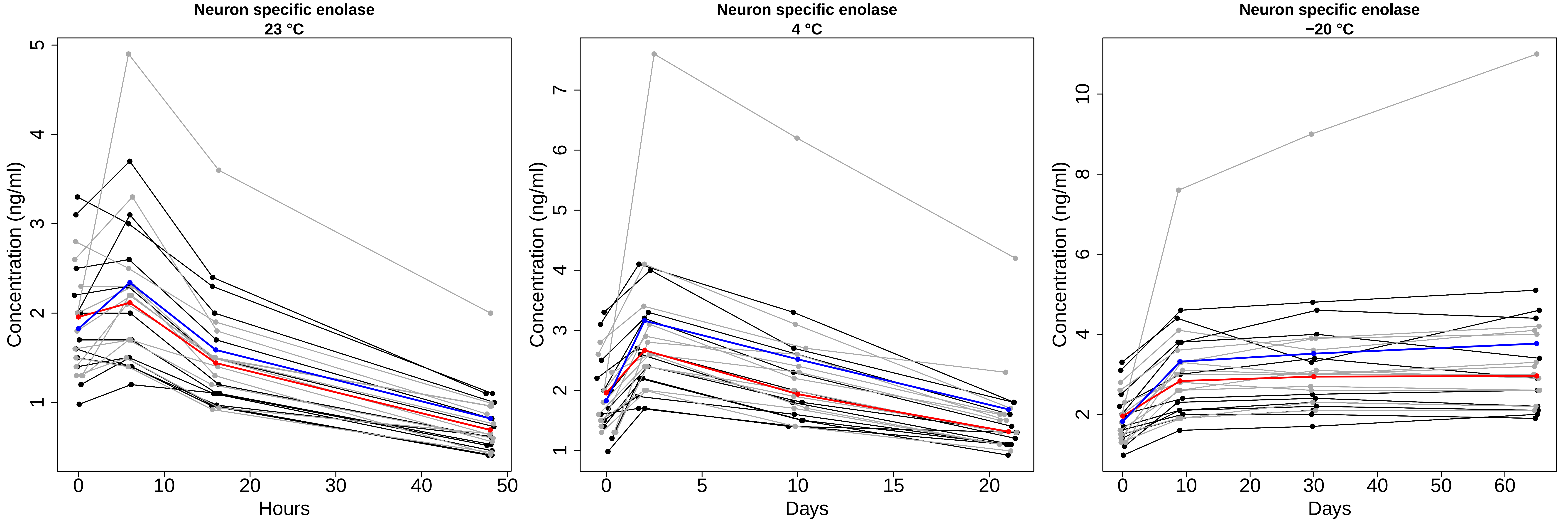
Neuron specific enolase
23 °C
Concentration (ng/ml)
Hours
Neuron specific enolase
4 °C
Concentration (ng/ml)
Days
Neuron specific enolase
−20 °C
Concentration (ng/ml)
Days

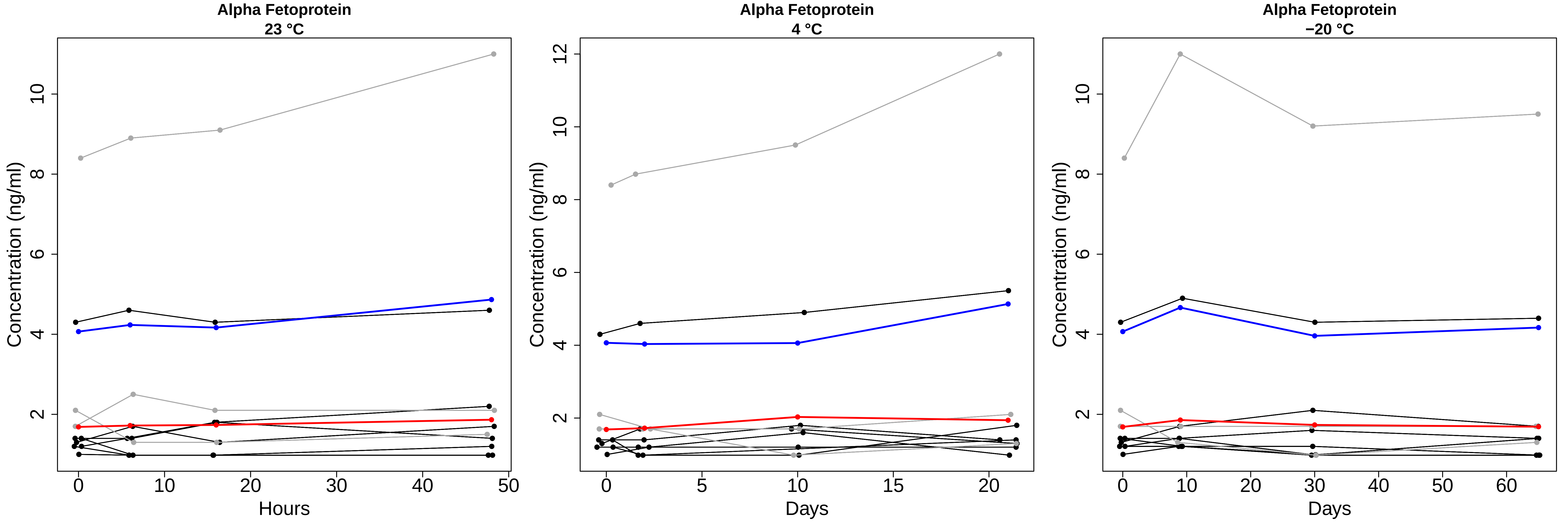

Alpha Fetoprotein
23 °C
Concentration (ng/ml)
Hours
Alpha Fetoprotein
4 °C
Concentration (ng/ml)
Days
Alpha Fetoprotein
−20 °C
Concentration (ng/ml)
Days

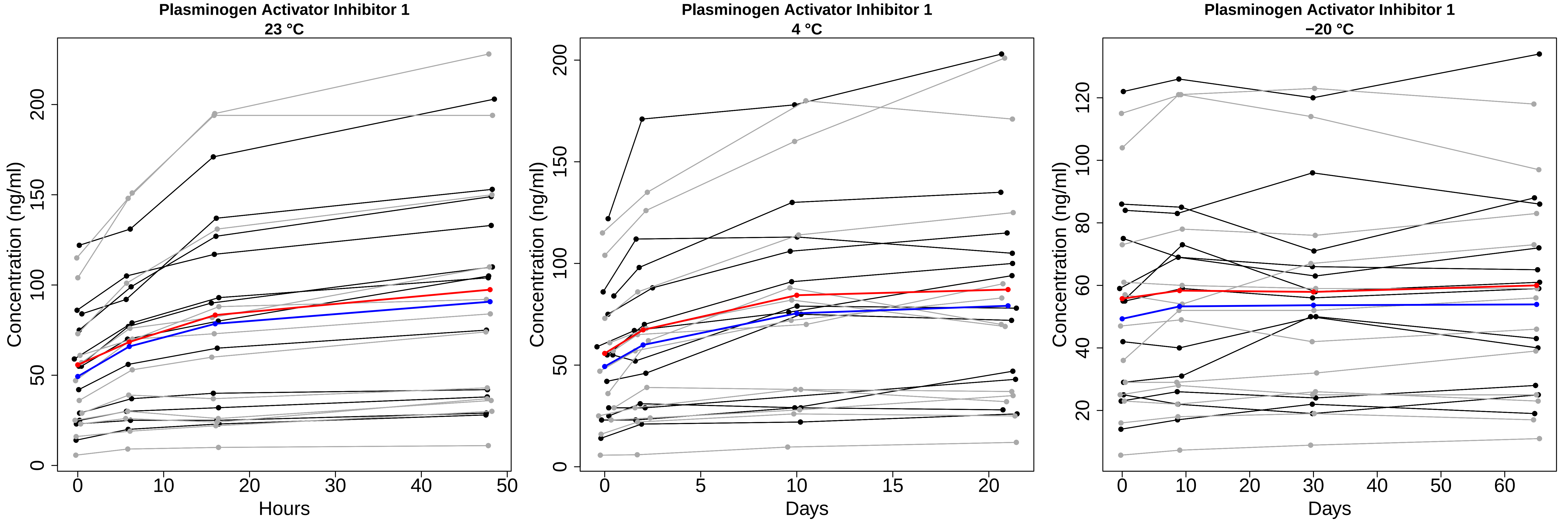

Plasminogen Activator Inhibitor 1
23 °C
Concentration (ng/ml)
Hours
Plasminogen Activator Inhibitor 1
4 °C
Concentration (ng/ml)
Days
Plasminogen Activator Inhibitor 1
−20 °C
Concentration (ng/ml)
Days

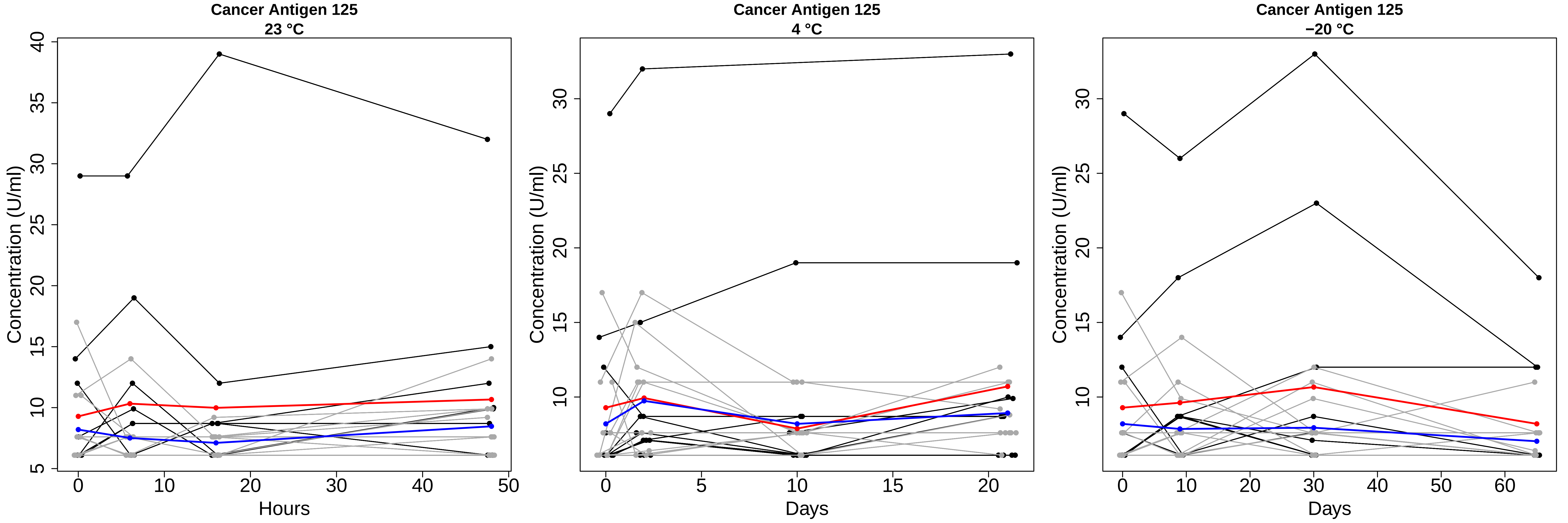

Cancer Antigen 125
23 °C
Concentration (U/ml)
Hours
Cancer Antigen 125
4 °C
Concentration (U/ml)
Days
Cancer Antigen 125
−20 °C
Concentration (U/ml)
Days

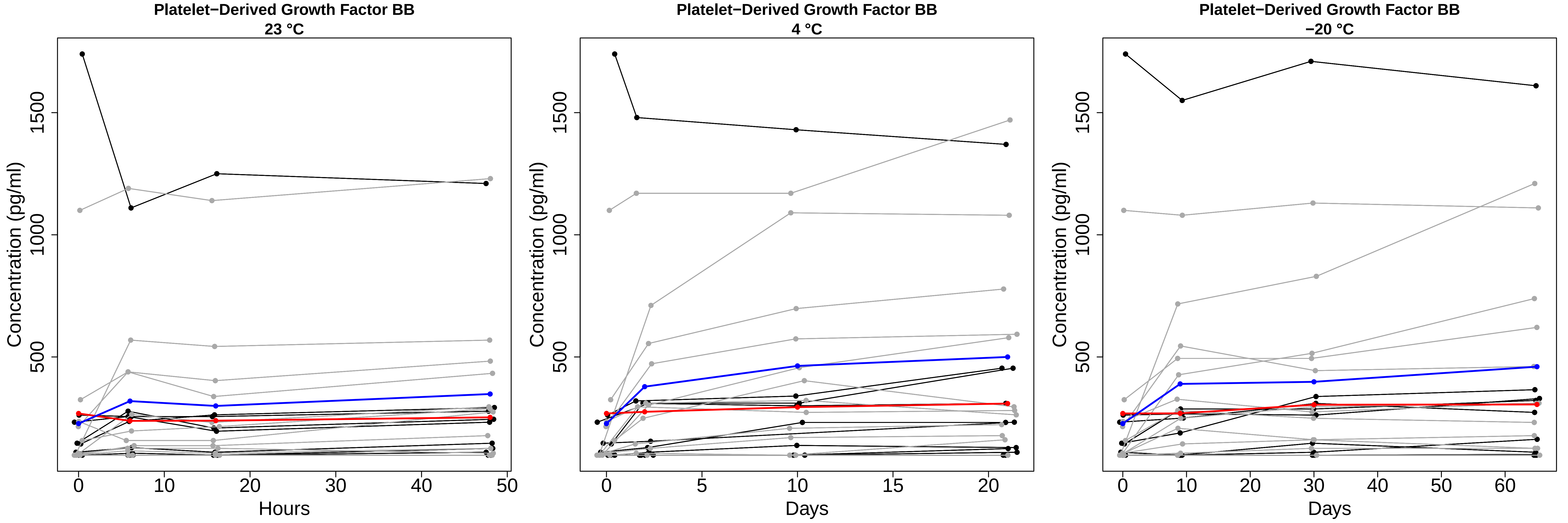

Platelet−Derived Growth Factor BB
23 °C
Concentration (pg/ml)
Hours
Platelet−Derived Growth Factor BB
4 °C
Concentration (pg/ml)
Days
Platelet−Derived Growth Factor BB
−20 °C
Concentration (pg/ml)
Days

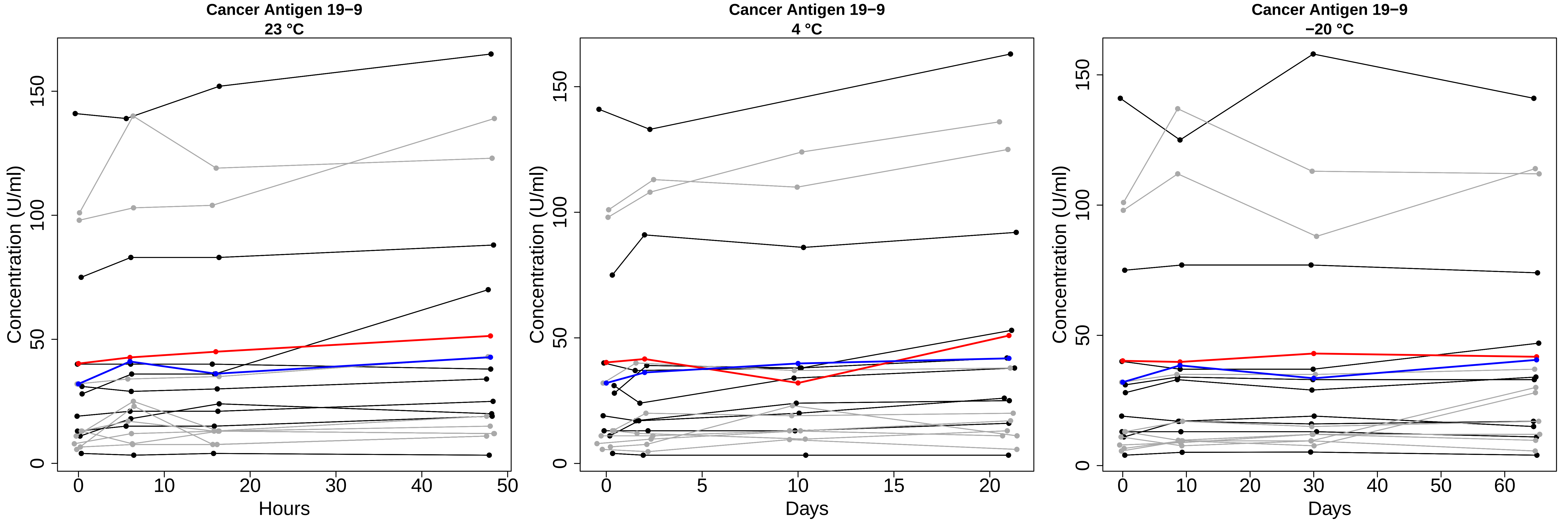

Cancer Antigen 19−9
23 °C
Concentration (U/ml)
Hours
Cancer Antigen 19−9
4 °C
Concentration (U/ml)
Days
Cancer Antigen 19−9
−20 °C
Concentration (U/ml)
Days

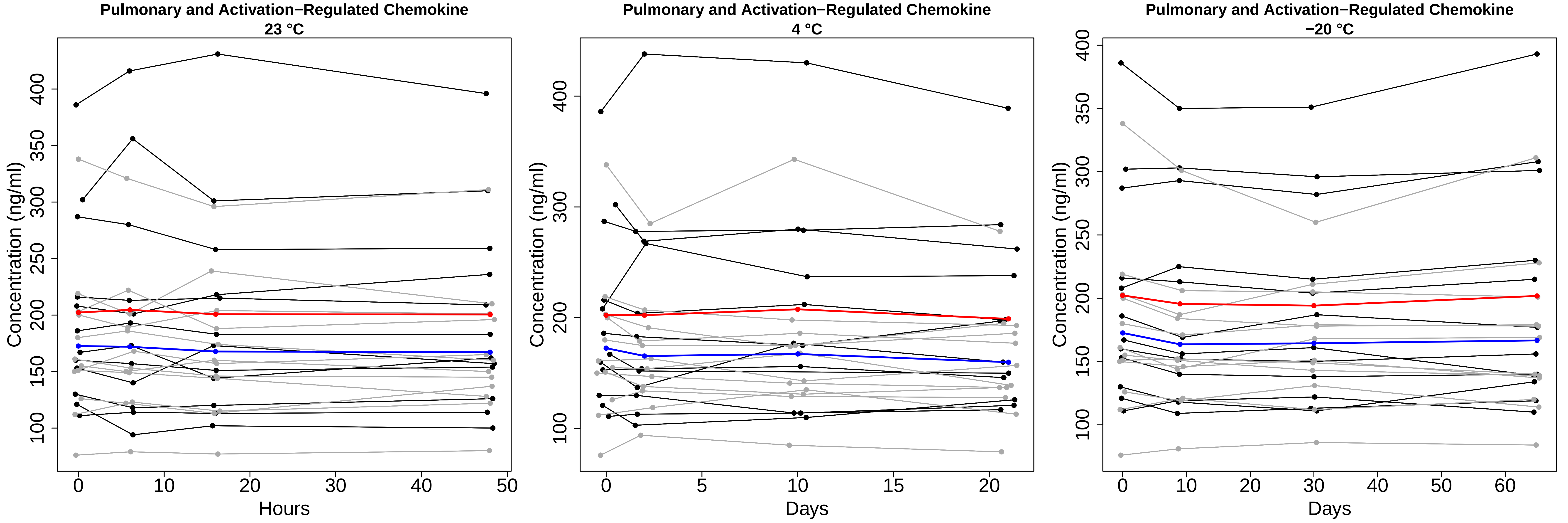

Pulmonary and Activation−Regulated Chemokine
23 °C
Concentration (ng/ml)
Hours
Pulmonary and Activation−Regulated Chemokine
4 °C
Concentration (ng/ml)
Days
Pulmonary and Activation−Regulated Chemokine
−20 °C
Concentration (ng/ml)
Days

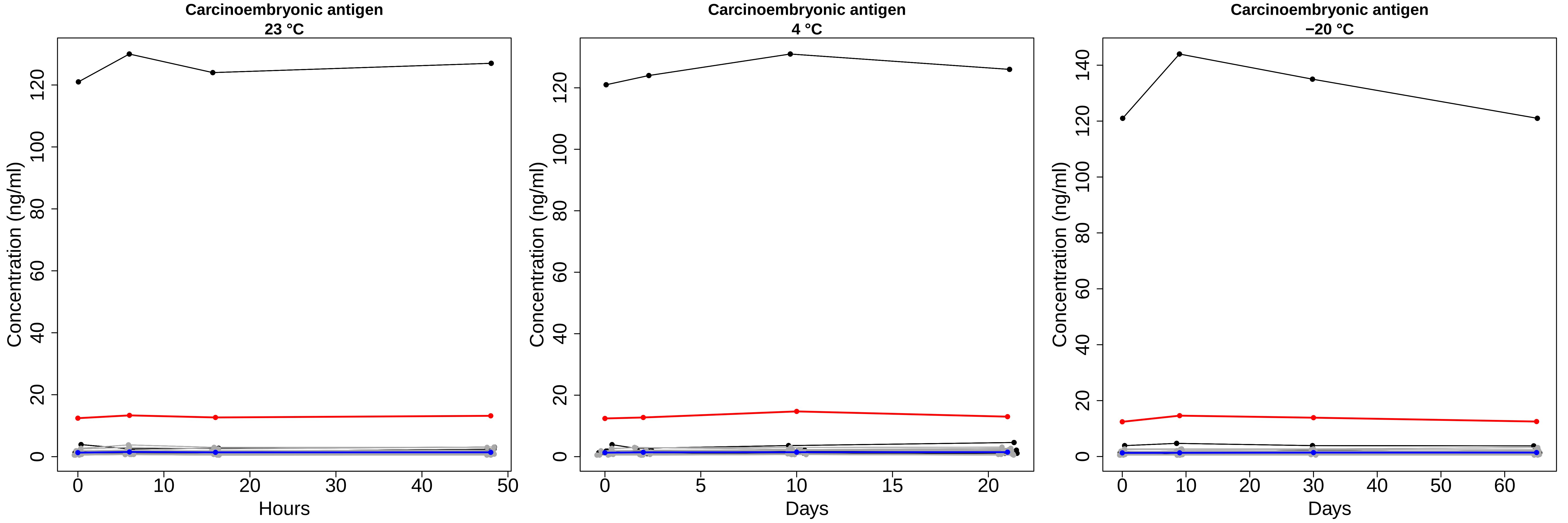
Carcinoembryonic antigen
23 °C
Concentration (ng/ml)
Hours
Carcinoembryonic antigen
4 °C
Concentration (ng/ml)
Days
Carcinoembryonic antigen
−20 °C
Concentration (ng/ml)
Days

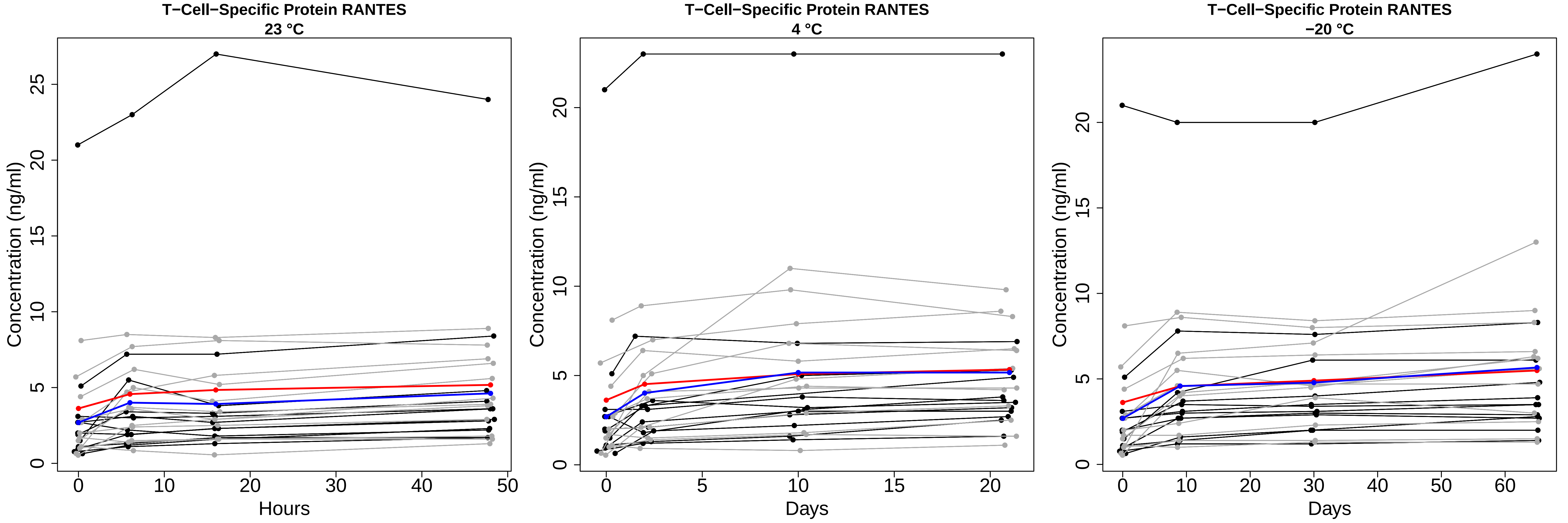
T−Cell−Specific Protein RANTES
23 °C
Concentration (ng/ml)
Hours
T−Cell−Specific Protein RANTES
4 °C
Concentration (ng/ml)
Days
T−Cell−Specific Protein RANTES
−20 °C
Concentration (ng/ml)
Days

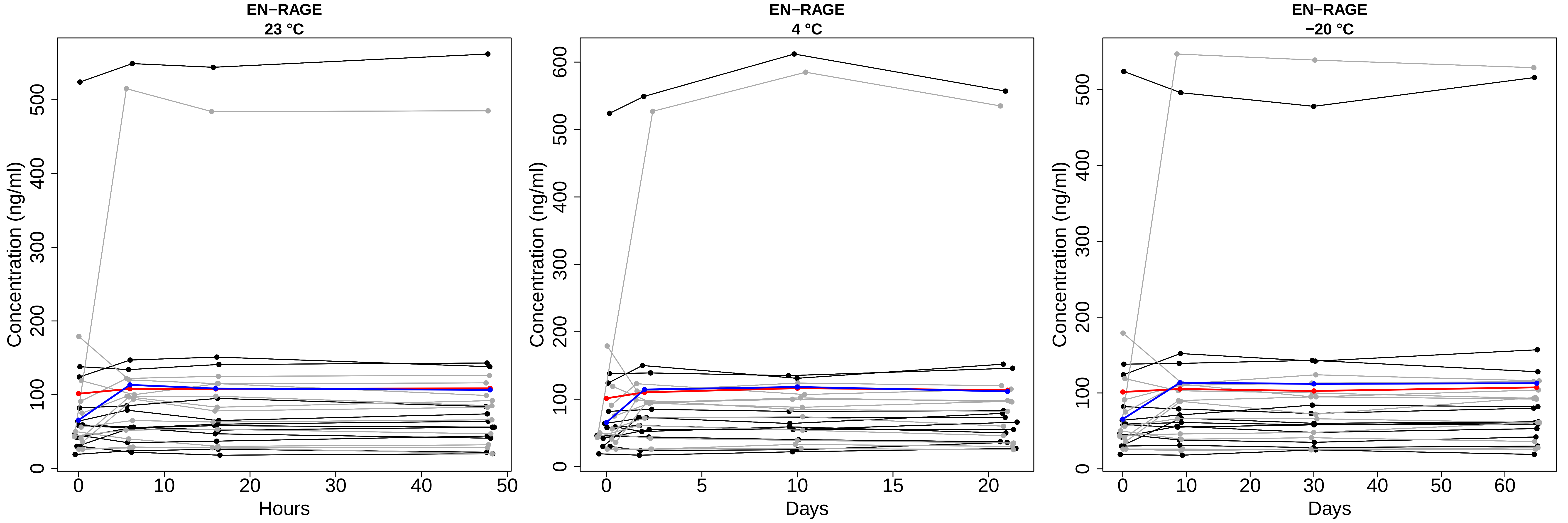

EN−RAGE
23 °C
Concentration (ng/ml)
Hours
0
100
200
300
400
500
0
10
20
30
40
50
EN−RAGE
4 °C
Concentration (ng/ml)
Days
0
100
200
300
400
500
600
0
5
10
15
20
EN−RAGE
−20 °C
Concentration (ng/ml)
Days
0
100
200
300
400
500
0
10
20
30
40
50
60

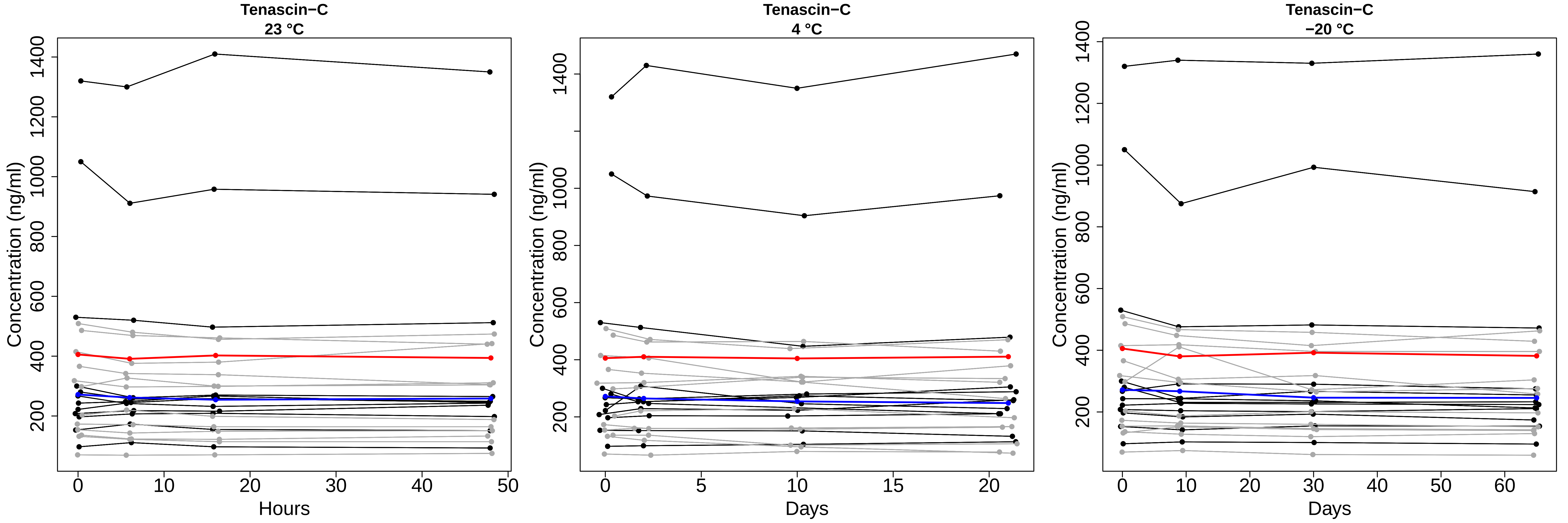
Tenascin−C
23 °C
Concentration (ng/ml)
Hours
Tenascin−C
4 °C
Concentration (ng/ml)
Days
Tenascin−C
−20 °C
Concentration (ng/ml)
Days

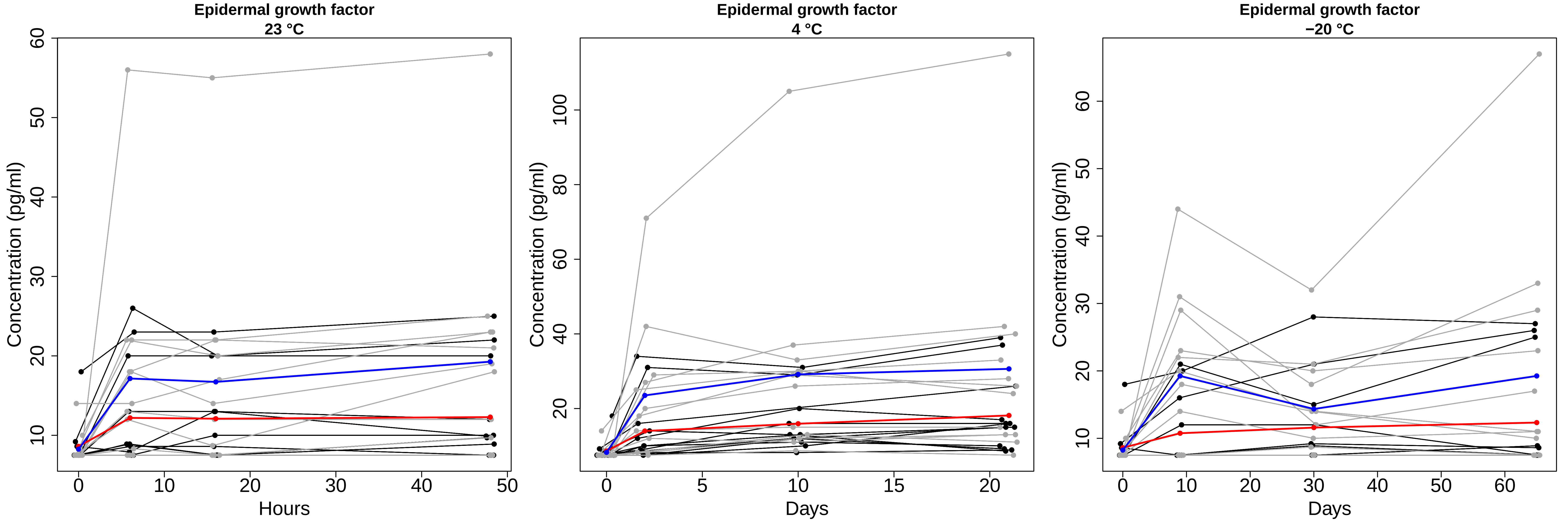

Epidermal growth factor
23 °C
Concentration (pg/ml)
Hours
0
10
20
30
40
50
60
Epidermal growth factor
4 °C
Concentration (pg/ml)
Days
0
5
10
15
20
40
60
80
100
Epidermal growth factor
−20 °C
Concentration (pg/ml)
Days
0
10
20
30
40
50
60

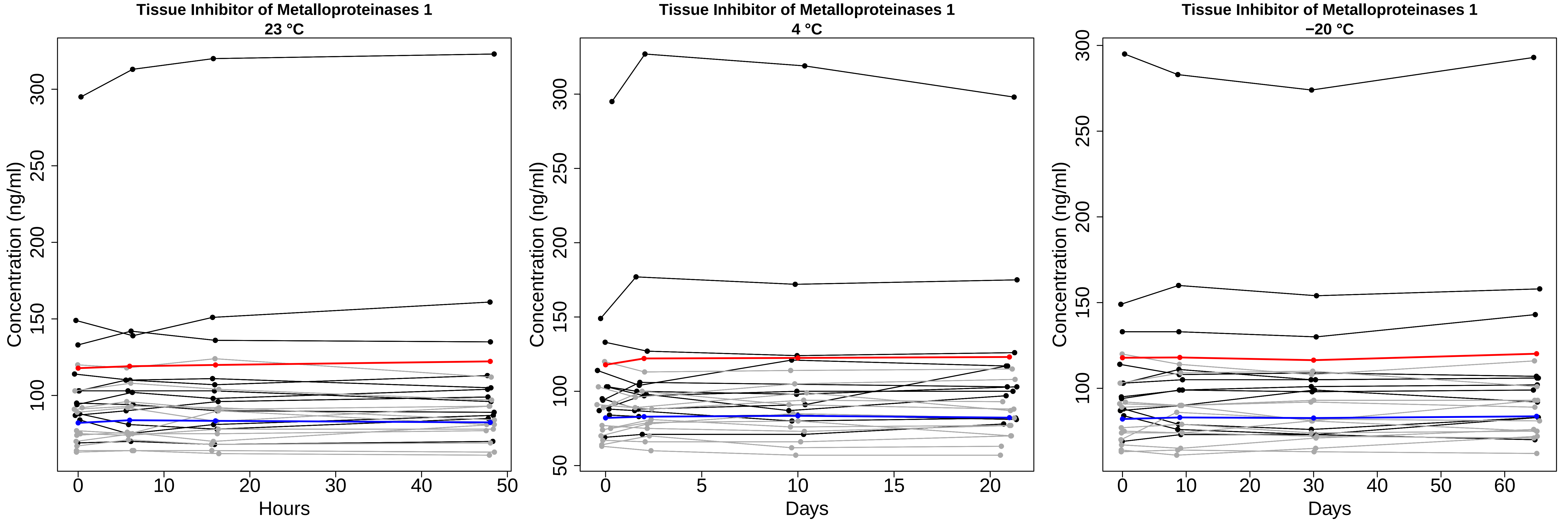

Tissue Inhibitor of Metalloproteinases 1
23 °C
Concentration (ng/ml)
Hours
Tissue Inhibitor of Metalloproteinases 1
4 °C
Concentration (ng/ml)
Days
Tissue Inhibitor of Metalloproteinases 1
−20 °C
Concentration (ng/ml)
Days

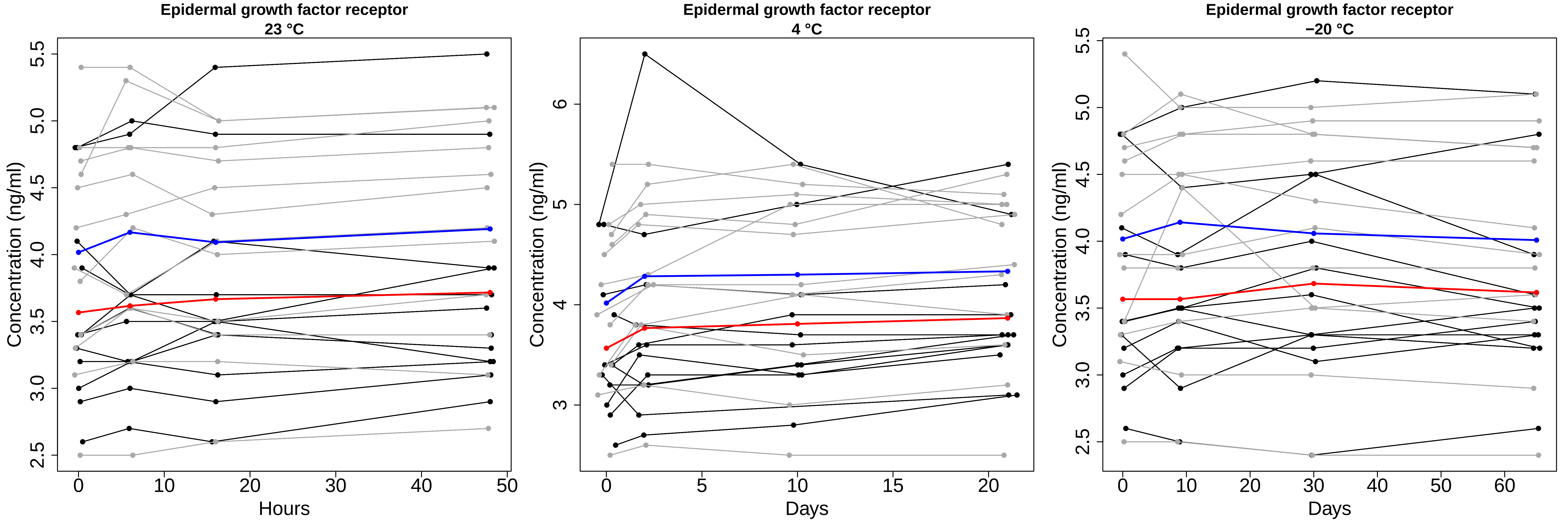

Epidermal growth factor receptor
23 °C
Concentration (ng/ml)
Hours
0
10
20
30
40
50
2.5
3.0
3.5
4.0
4.5
5.0
5.5
Epidermal growth factor receptor
4 °C
Concentration (ng/ml)
Days
0
5
10
15
20
3
4
5
6
Epidermal growth factor receptor
−20 °C
Concentration (ng/ml)
Days
0
10
20
30
40
50
60
2.5
3.0
3.5
4.0
4.5
5.0
5.5

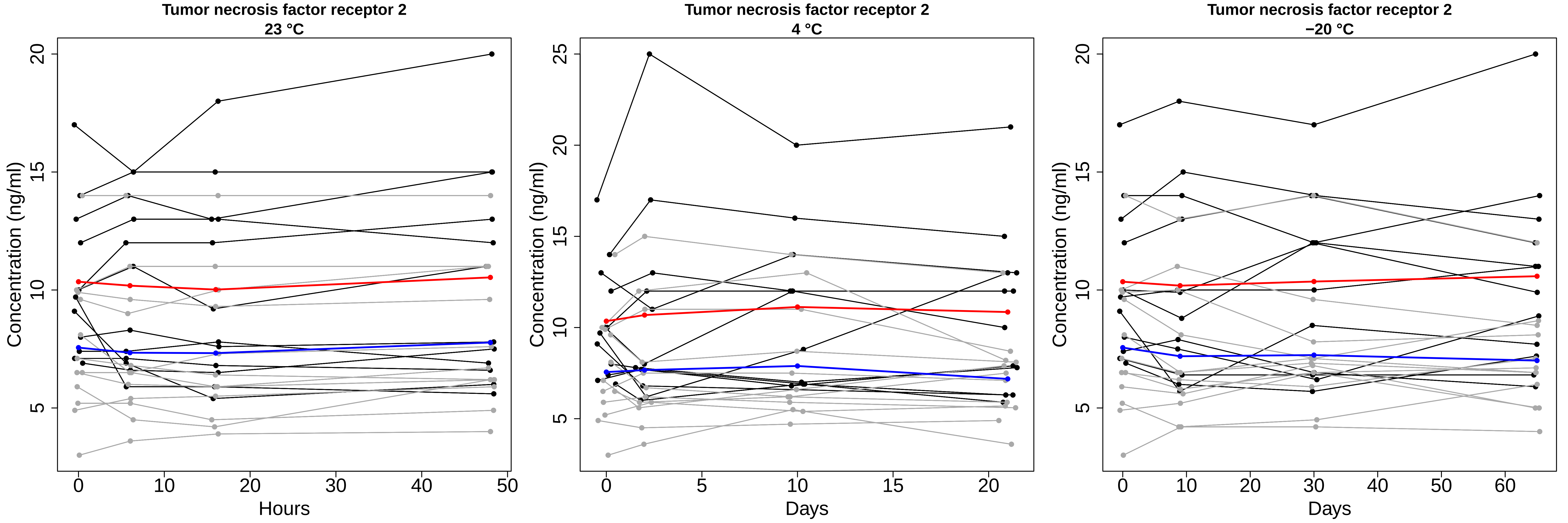
Tumor necrosis factor receptor 2
23 °C
Concentration (ng/ml)
Hours
Tumor necrosis factor receptor 2
4 °C
Concentration (ng/ml)
Days
Tumor necrosis factor receptor 2
−20 °C
Concentration (ng/ml)
Days

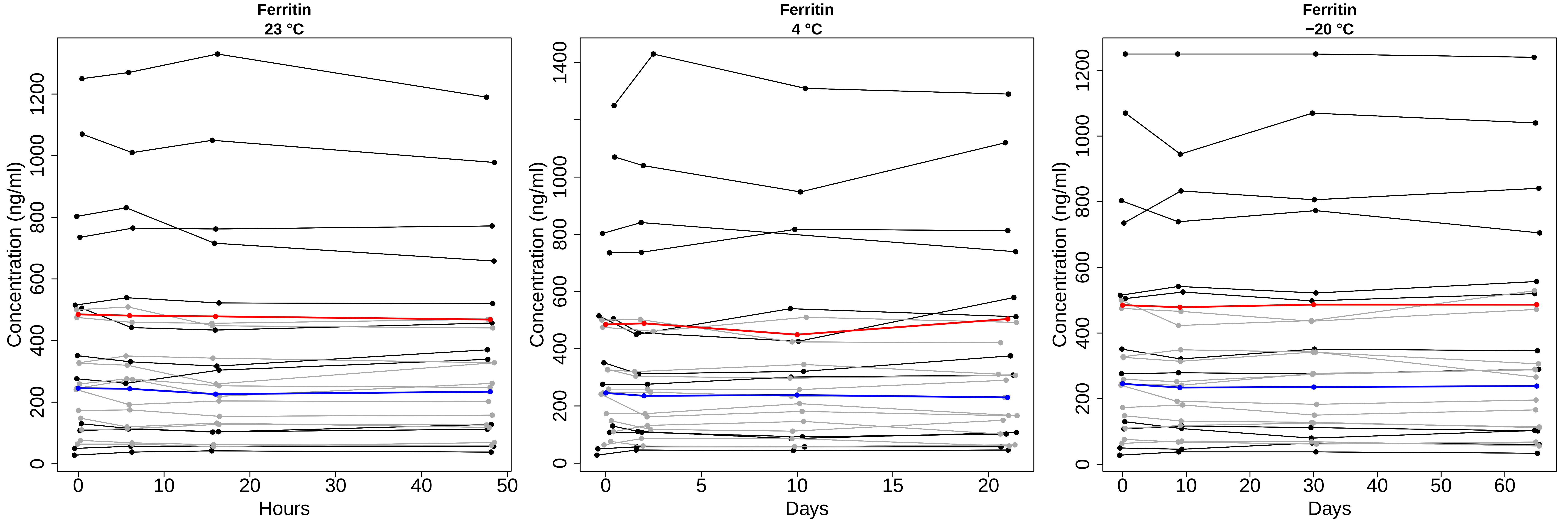
Ferritin
23 °C
Ferritin
4 °C
Ferritin
−20 °C
Concentration (ng/ml)
Hours
Days
Days
0
200
400
600
800
1000
1200
1400
0
10
20
30
40
50
5
15
60

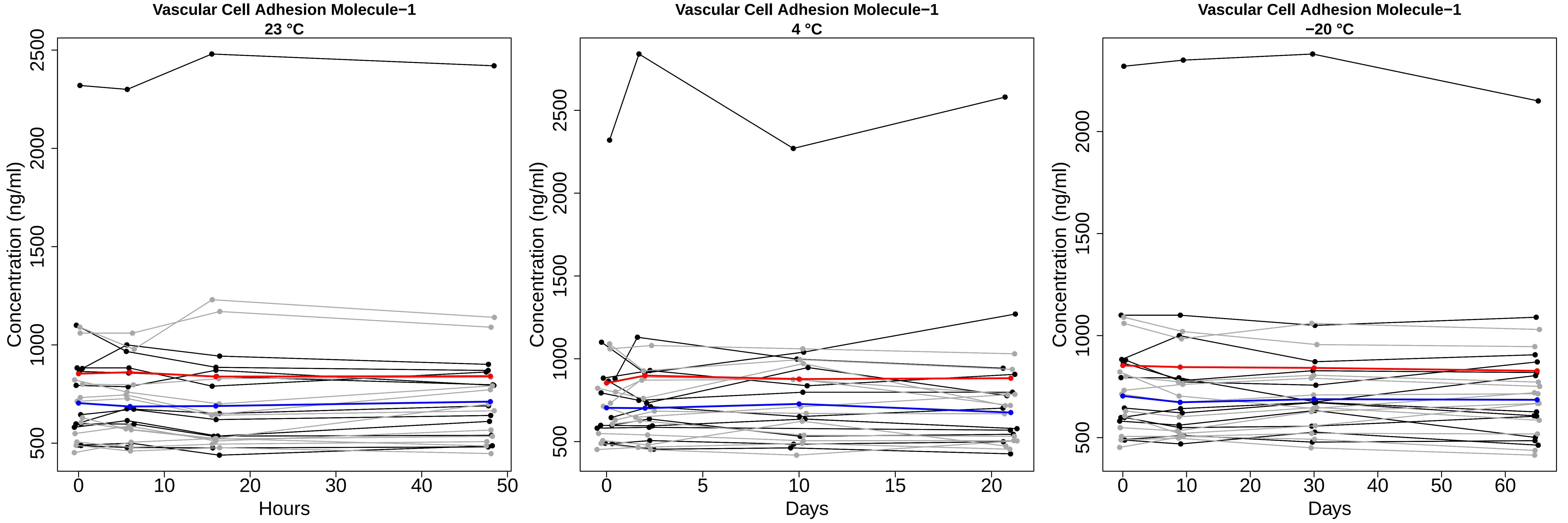
Vascular Cell Adhesion Molecule−1
23 °C
Concentration (ng/ml)
Hours
Vascular Cell Adhesion Molecule−1
4 °C
Concentration (ng/ml)
Days
Vascular Cell Adhesion Molecule−1
−20 °C
Concentration (ng/ml)
Days

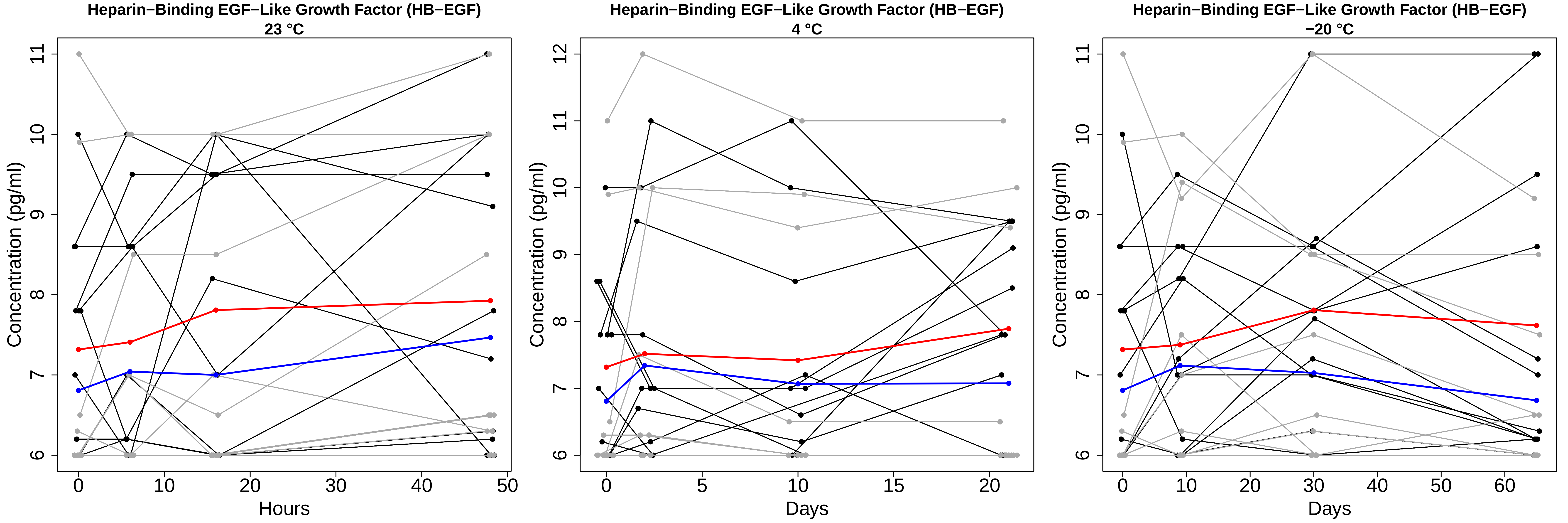
Heparin−Binding EGF−Like Growth Factor (HB−EGF)
23 °C
Concentration (pg/ml)
Hours
Heparin−Binding EGF−Like Growth Factor (HB−EGF)
4 °C
Concentration (pg/ml)
Days
Heparin−Binding EGF−Like Growth Factor (HB−EGF)
−20 °C
Concentration (pg/ml)
Days

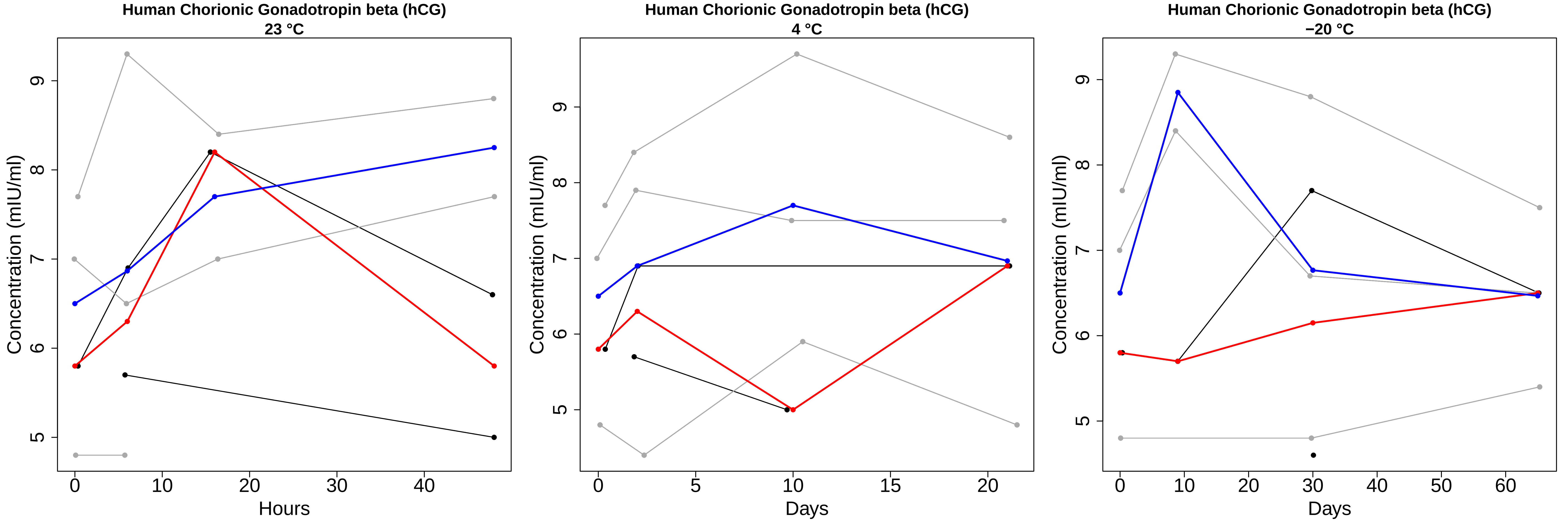
Human Chorionic Gonadotropin beta (hCG)
23 °C
Concentration (mIU/ml)
Hours
Human Chorionic Gonadotropin beta (hCG)
4 °C
Concentration (mIU/ml)
Days
Human Chorionic Gonadotropin beta (hCG)
−20 °C
Concentration (mIU/ml)
Days

Figure S3: Time courses for clinical proteins in K_2EDTA plasma at all temperatures. Longitudinal results for individual patients/donors are shown. Blue points & lines represent the mean of the cancer-free control donors while grey points & lines represent the individuals; red points & lines represent the mean of the GI cancer patients while black points & lines represent the individuals.

Table S1. Clinically relevant proteins that were quantified in K_2EDTA plasma samples using the Luminex bead based multiplex immunoassay, their numbers of *free* cysteine and methionine residues, and the clonality of capture and detection antibodies employed.

Protein	Clinical Utility	Number of Free Cysteine Residues	Number of Methionine Residues	Capture Antibody Clonality	Detection Antibody Clonality
Adiponectin	Biomarker for obesity-related diseases, i.e., metabolic syndromes and coronary artery disease	1-2	4	monoclonal	monoclonal
Alpha-2-Macroglobulin (A2M)	Biomarker for kidney and liver diseases	0	25	polyclonal	- [a]
Alpha-Fetoprotein (AFP)	Cancer Biomarker	2	8	monoclonal	monoclonal
Cancer Antigen 125 (CA-125)	Cancer Biomarker	0-29	434	monoclonal	monoclonal
Cancer Antigen 19-9 (CA-19-9)	Cancer Biomarker	N/A [b]	N/A [b]	monoclonal	monoclonal
Carcinoembryonic Antigen (CEA)	Cancer Biomarker	0-10	0	monoclonal	monoclonal
EN-RAGE	Marker for inflammation/Immune responses	0	0	polyclonal	polyclonal
Epidermal Growth Factor (EGF)	Cancer Biomarker	0	1	monoclonal	polyclonal
Epidermal Growth Factor Receptor (EGFR)	Cancer Biomarker	0	10	monoclonal	monoclonal
Ferritin (FRTN)	Cancer and inflammatory response marker	3 in heavy chain; 1 in light chain	4 per chain	monoclonal	polyclonal
Heparin-Binding EGF-Like Growth Factor (HB-EGF)	Cancer Biomarker	0	0	polyclonal	polyclonal

Myoglobin	Marker for cardiac and vascular diseases, cancer, and renal function/toxicity	1	3	monoclonal	monoclonal
Neuron Specific Enolase (NSE)	Cancer Biomarker	6	8	monoclonal	monoclonal
Plasminogen Activator Inhibitor 1 (PAI-1)	Marker for cardiac and vascular diseases, neurological diseases, and cancer	0	16	polyclonal	polyclonal
Platelet-Derived Growth Factor BB (PDGF-BB)	Cancer Biomarker	0	1	monoclonal	monoclonal
Pulmonary and Activation-Regulated Chemokine (PARC)	Marker for cardiac and vascular diseases and inflammatory/immune response	0	0	polyclonal	polyclonal
T-Cell-Specific Protein RANTES (RANTES)	Marker for neurological diseases and inflammatory/immune response	0	1	monoclonal	polyclonal
Tenascin-C	Cancer Biomarker	0-14	21	monoclonal	monoclonal
Tissue Inhibitor of Metalloproteinases 1 (TIMP-1)	Marker for cardiac and vascular diseases and inflammatory/immune response	0	3	monoclonal	polyclonal
Tumor necrosis factor receptor 2 (TNFR2)	Marker for cancer and inflammation/Immune responses	0-2	4	monoclonal	polyclonal
Vascular Cell Adhesion Molecule-1 (VCAM-1)	Marker for cardiac and vascular diseases, neurological diseases, cancer, autoimmune diseases, and inflammatory/ immune response	0-7	14	polyclonal	polyclonal

[a] A competitive assay was used for detection.

[b] Not applicable. CA 19-9 is the sialyl-Lewis^{A} tetrasaccharide that exists on more than one unique protein.

Table S2. Linear regression analysis results for baseline concentrations of clinical proteins with patient age. Statistically insignificant associations are shown in grey font.

Protein	Slope	Slope Stnd. Error	p-value	Bonferroni-Adjusted p-value
Adiponectin (ug/ml)	0.00833	0.0079	7.9E-12	1.7E-10
Myoglobin (ng/ml)	0.02196	0.0118	3.9E-15	8.2E-14
Alpha-2-Macroglobulin (mg/ml)	0.01139	0.0031	1.2E-14	2.5E-13
Neuron specific enolase (ng/ml)	0.00822	0.0042	4.7E-09	9.9E-08
Alpha Fetoprotein (ng/ml)	9.11E-04	0.0076	3.3E-02	6.9E-01
Plasminogen Activator Inhibitor 1 (ng/ml)	-0.00899	0.0106	5.4E-18	1.1E-16
Cancer Antigen 125 (U/ml)	0.01051	0.0055	5.9E-18	1.2E-16
Platelet-Derived Growth Factor BB (pg/ml)	0.01006	0.0109	1.3E-20	2.8E-19
Cancer Antigen 19-9 (U/ml)	0.03497	0.0160	4.3E-10	9.0E-09
Pulmonary and Activation-Regulated Chemokine (ng/ml)	0.01149	0.0049	7.7E-28	1.6E-26
Carcinoembryonic antigen (ng/ml)	0.03175	0.0144	1.3E-01	1.0E+00
T-Cell-Specific Protein RANTES (ng/ml)	0.01205	0.0124	6.9E-04	1.4E-02
EN-RAGE (ng/ml)	-0.00861	0.0104	5.3E-19	1.1E-17
Tenascin-C (ng/ml)	-8.35E-05	0.0099	1.1E-22	2.2E-21
Epidermal growth factor (pg/ml)	-8.45E-04	0.0032	2.5E-24	5.1E-23
Tissue Inhibitor of Metalloproteinases 1 (ng/ml)	0.00739	0.0045	7.4E-28	1.6E-26
Epidermal growth factor receptor (ng/ml)	-0.00707	0.0026	3.7E-20	7.8E-19
Tumor necrosis factor receptor 2 (ng/ml)	0.01243	0.0049	7.2E-19	1.5E-17
Ferritin (ng/ml)	-0.01253	0.0139	4.7E-19	9.9E-18
Vascular Cell Adhesion Molecule-1 (ng/ml)	0.00954	0.0048	1.2E-30	2.6E-29
Heparin-Binding EGF-Like Growth Factor (HB-EGF) (pg/ml)	0.00578	0.0025	1.2E-24	2.6E-23

References Cited in Supplemental Data

22. Jeffs, J. W., Jehanathan, N., Thibert, S. M. F., Ferdosi, S., Pham, L., Wilson, Z. T., Breburda, C., and Borges, C. R. (2019) Delta-S-Cys-Albumin: A Lab Test that Quantifies Cumulative Exposure of Archived Human Blood Plasma and Serum Samples to Thawed Conditions. *Mol Cell Proteomics* 18, 2121-2137.
